# Profiling Nonrecipients of Mass Drug Administration for Schistosomiasis and Hookworm Infections: A Comprehensive Analysis of Praziquantel and Albendazole Coverage in Community-Directed Treatment in Uganda

**DOI:** 10.1093/cid/civ829

**Published:** 2015-09-25

**Authors:** Goylette F. Chami, Andreas A. Kontoleon, Erwin Bulte, Alan Fenwick, Narcis B. Kabatereine, Edridah M. Tukahebwa, David W. Dunne

**Affiliations:** 1 Departments of Land Economy; 2 Pathology, University of Cambridge; 3 Schistosomiasis Control Initiative, Imperial College London, United Kingdom; 4 Development Economics Group, Wageningen University, The Netherlands; 5 Bilharzia and Worm Control Programme, Vector Control Division, Uganda Ministry of Health, Kampala

**Keywords:** mass drug administration, coverage, schistosomiasis, soil-transmitted helminths, sub-Saharan Africa

## Abstract

***Background.*** Repeated mass drug administration (MDA) with preventive chemotherapies is the mainstay of morbidity control for schistosomiasis and soil-transmitted helminths, yet the World Health Organization recently reported that less than one-third of individuals who required preventive chemotherapies received treatment.

***Methods.*** Coverage of community-directed treatment with praziquantel (PZQ) and albendazole (ALB) was analyzed in 17 villages of Mayuge District, Uganda. National drug registers, household questionnaires, and parasitological surveys were collected to track 935 individuals before and after MDA. Multilevel logistic regressions, including household and village effects, were specified with a comprehensive set of socioeconomic and parasitological variables. The factors predicting who did not receive PZQ and ALB from community medicine distributors were identified.

***Results.*** Drug receipt was correlated among members within a household, and nonrecipients of PZQ or ALB were profiled by household-level socioeconomic factors. Individuals were less likely to receive either PZQ or ALB if they had a Muslim household head or low home quality, belonged to the minority tribe, or had settled for more years in their village. Untreated individuals were also more likely to belong to households that did not purify drinking water, had no home latrine, and had no members who were part of the village government.

***Conclusions.*** The findings demonstrate how to locate and target individuals who are not treated in MDA. Infection risk factors were not informative. In particular, age, gender, and occupation were unable to identify non-recipients, although World Health Organization guidelines rely on these factors. Individuals of low socioeconomic status, minority religions, and minority tribes can be targeted to expand MDA coverage.

## Abstract (precis)

We tracked individuals during mass drug administration. Untreated individuals were not hard to reach for local drug distributors and would comply with treatment if offered. Household socioeconomic status and minority group affiliation identified untreated individuals; infection risk factors were not predictive of drug receipt.

**(See the Editorial Commentary by King on pages 208–9.)**

An estimated 207 and 576 million individuals, respectively, are infected with *Schistosoma* and hookworm species [1, 2]. The majority of schistosomiasis infections occur in childhood and, if left untreated, can cause irreversible disease in adulthood [1]. These diseases include portal hypertension (caused by *Schistosoma mansoni* or *Schistosoma japonicum*) and bladder squamous cell carcinoma (caused by *Schistosoma haematobium*). Heavy hookworm infection intensity, which can be found in children but is most common in adults, can lead to iron deficiency anemia, protein malnutrition, and diarrhea [2].

Mass drug administration (MDA) is the mainstay of morbidity control for human helminthiases [3]. MDA is the delivery, en masse, of free single-treatment preventive chemotherapies (PCs) in regular intervals to endemic populations. PCs are “preventive’” in that they successfully curtail morbidity and are safe to administer to uninfected individuals. Endemic areas are classified by rapid sampling, usually of children in primary schools, to assess infection prevalence. Schistosomiasis is treated with praziquantel (PZQ), and soil-transmitted helminths (STHs) are treated with albendazole (ALB) or mebendazole. Repeated annual or biannual treatments are necessary mainly because of susceptibility to reinfection after treatment [4, 5]. Coverage (the proportion of persons requiring and receiving PCs through MDA) was an estimated 16.88% among all individuals requiring treatment for schistosomiasis in 2012 [6] and 30.63% among children needing PCs for STHs in 2011 [7].

In this article, we identify the factors that affect access to PCs during MDA. We focus on community-directed treatment (CDT), the only mode of MDA delivery of PZQ and ALB to both adults and children [8–10]. CDT involves 2 locally selected community medicine distributors (CMDs), who are trained annually by a district health officer (DHO) to administer correct treatment dosages, determine ineligibility of individuals, and record these treatments in national drug registers [3]. Apart from receiving remuneration for training, CMDs are unpaid volunteers.

A nonrecipient is someone who was not approached by the CMD or was offered treatment and deliberately refused (noncompliance) [11]. Individuals who are not approached by CMDs often include hard-to-reach or mobile populations, whereas noncompliance has been associated with a fear of adverse effects, social differences between the recipient and CMD, and a lack of health education [11, 12]. In sub-Saharan Africa, rates of compliance with ivermectin (IVM) CDT for onchocerciasis and lymphatic filariasis was higher among men, certain ethnic groups, Christians, and landowners [13–17]. For schistosomiasis infections in Uganda, qualitative analyses [11, 18] suggested that at-risk fishermen and adults did not receive PZQ treatment. These profiles of CDT nonrecipients have been poorly and incompletely described. Analyses of PZQ receipt [11, 18] relied on qualitative accounts of special interest groups, and studies of IVM distribution assessed only noncompliance [13–17].

This article presents what is to our knowledge the first quantitative assessment of nonrecipients of CDT for schistosomiasis and STHs. Research was conducted in Uganda, the first country in sub-Saharan Africa to adopt MDA for schistosomiasis in 2003 and to integrate control measures with STHs in 2004 [19, 20]. National drug registers, household questionnaires, and parasitological surveys were collected to determine the observable factors that identify and predict which individuals do not receive PZQ or ALB treatment from CMDs.

## METHODS

### Study Location and Participant Selection

This study was conducted from August-November 2013 in the context of a routine national helminth control program. Seventeen villages were surveyed within 5 kilometers of Lake Victoria in Mayuge District, Uganda (see Supplementary Text for village selection). This area is endemic only for intestinal schistosomiasis (*S. mansoni*), and the species of hookworm is unknown. In these villages, 517 households and 1034 participants were selected by CMDs, and ≥30 households within each village were sampled. Researchers provided CMDs with stratified sampling instructions to sample 1 adult and 1 child from each home (see Supplementary Text for additional details). Paired sampling was used to determine whether CMDs distributed treatments from home to home as trained.

### Data Sources

**Figure 1.**
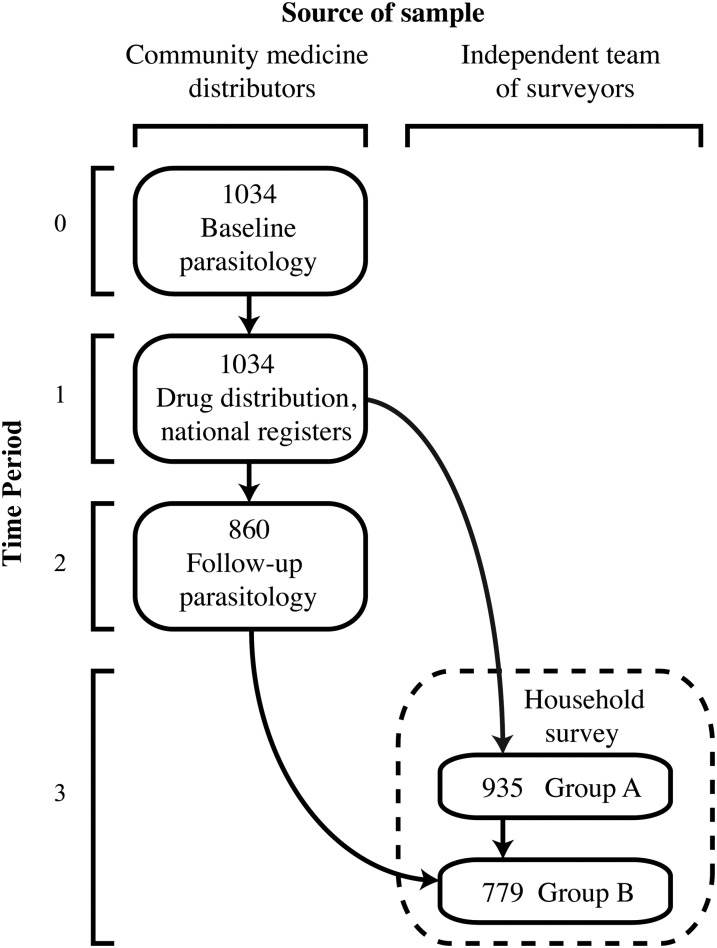
Chronology of data collected and schematic of analysis completed. Data were collected over 4 time periods for the initial 1034 participants who provided stool samples for baseline parasitology. The source of the initial sampling or retrieval of these participants was the village community medicine distributor or an independent team of surveyors. Not all data sources were available for the initial 1034 participants. Refer to “Methods” section for a full explanation.

Figure 1 presents the chronology of data collected before and after MDA for the 1034 study participants. In August–September 2013, data were first collected to measure the baseline (pretreatment) infection intensities of the study participants. CMDs recruited participants without the presence of researchers, which ensured that participants were identifiable and not hard for CMDs to reach. Each participant provided 1 stool sample, and standard thick-smear (41.7 mg) Kato-Katz methods [21] were used to count hookworm eggs (within 30 minutes of preparation) and *S. mansoni* eggs (24 hours after preparation) for 2 slides. At this time, CMDs were not informed of forthcoming MDA or research activities.

During the second period of this study, MDA was ongoing. In the last 2 weeks of September, the DHO trained CMDs and provided PZQ, ALB, IVM, and new national drug registers. The DHO instructed CMDs to deliver PZQ in the first week of October, followed by a package of ALB and IVM (for lymphatic filariasis) in the second week. The DHO was able to confirm that all CMDs completed their training, received enough pills to treat all eligible persons in their village, and knew of all the homes in their village. To avoid national and district administrative inefficiencies, the DHO was provided with a car to enable the timely completion of CMD training. All CMDs were trained and ready to begin MDA by 1 October 2013. No instruction was given to CMDs by the DHO to treat the study participants, and researchers were not present during any MDA activities.

After MDA, follow-up parasitology and household questionnaires were conducted. Villages were visited beginning 1 November 2013. CMDs were told that the purpose of these surprise visits was to examine drug efficacy from the follow-up parasitology, and laboratory technicians would need to know which participants received PZQ or ALB. A list of the initial 1034 participants was provided to CMDs, who were able to retrieve 860 of these individuals to provide a second stool sample. A researcher also inspected the national registers with the CMDs and recorded which of the initial 1034 participants received PZQ, ALB, or IVM. CMDs were involved only in research activities that reflect routine practices in MDA. Accordingly, after the second round of parasitology, independent teams of local villagers were used to conduct household surveys in their respective villages. These surveys gathered socioeconomic information on 935 of the 1034 initial participants (group A in Figure 1). Among these 935 individuals, 779 were included in the group of 860 individuals who provided a follow-up stool sample (group B in Figure 1). No differences in baseline infection intensity, age, or sex were found between individuals included in the household survey and those who were not interviewed (Supplementary Table 1).

### Variables

Infection intensity was calculated as eggs per gram. Egg counts for each slide (41.7 mg) were multiplied by 24 and averaged to determine eggs per gram. Individuals with ≥1 detectable egg per gram were classified as infected. Baseline village prevalence was calculated as the proportion of infected individuals from the initial 1034 participants. PZQ, ALB, and IVM treatments are represented as binary variables; these indicators are positive if the CMD recorded an individual as receiving the drug in the national register. Socioeconomic variables were included from the household survey to capture social status, wealth, demographics, and other observable characteristics of individuals (see Supplementary Text for full variable definitions). Table 1 presents a summary of all variables.

**Table 1.** Descriptive Characteristics of Study Participants^a^

Variable	Full Sample (n = 935)	PZQ Recipients (n = 492)	PZQ Nonrecipients (n = 443)	ALB Recipients (n = 384)	ALB Nonrecipients (n = 551)
Baseline EPG, mean (SD)^b^					
*Schistosoma mansoni*	219.7 (870.4)	149.8 (598.8)	297.3 (1091.4)	158.6 (610.9)	262.3 (1011.1)
Hookworm	348.6 (1486.2)	254.1 (1107.7)	453.6 (1812.0)	262.5 (1110.5)	408.5 (1698.0)
Age, mean (SD)	24.2 (16.5)	24.5 (16.5)	23.8 (16.5)	24.8 (16.4)	23.7 (16.7)
Female, No. (%)	564 (60.3)	286 (58.1)	278 (62.8)	232 (60.4)	332 (60.3)
Educational level, mean (SD)^c^	3.5 (3.0)	3.5 (3.1)	3.5 (2.9)	3.4 (3.1)	3.5 (2.9)
Occupation, No. (%)^d^					
No income-earning occupation	607 (64.9)	316 (64.2)	291 (65.7)	242 (63.0)	365 (66.2)
Fisherman or fishmonger	41 (4.4)	21 (4.3)	20 (4.5)	17 (4.4)	24 (4.4)
Business owner	25 (2.7)	10 (2.0)	15 (3.4)	12 (3.1)	13 (2.4)
Rice farmer	36 (3.9)	8 (1.6)	28 (6.3)	9 (2.3)	27 (4.9)
Other farmer	173 (18.5)	102 (20.7)	71 (16.0)	75 (19.5)	98 (17.8)
Schoolteacher	10 (1.1)	5 (1.0)	5 (1.1)	4 (1.0)	6 (1.1)
Health worker	6 (0.6)	6 (1.2)	0 (0)	5 (1.3)	1 (0.2)
Other	37 (4.0)	24 (4.9)	13 (2.9)	20 (5.2)	17 (3.1)
Muslim household head, No. (%)	275 (29.4)	121 (24.6)	154 (34.8)	97 (25.3)	178 (32.3)
Household head belongs to village majority tribe, No. (%)	500 (53.5)	294 (59.8)	206 (46.5)	219 (57.0)	281 (51.0)
Time household settled in village, mean (SD), y	16.7 (11.5)	17.4 (11.3)	16.0 (11.7)	16.2 (11.0)	17.1 (11.9)
Home quality score, mean (SD)^e^	7.4 (3.3)	7.9 (3.2)	6.7 (3.3)	7.9 (3.3)	7.0 (3.2)
Households, No. (%)					
With purified drinking water	400 (42.8)	221 (44.9)	179 (40.4)	177 (46.1)	223 (40.5)
With no home latrine	60 (6.4)	23 (4.7)	37 (8.4)	15 (3.9)	45 (8.2)
Including former or current village chairman	44 (4.7)	29 (5.9)	15 (3.4)	25 (6.5)	19 (3.4)
Including other former or current village government member	81 (8.7)	52 (10.6)	29 (6.5)	39 (10.2)	42 (7.6)
Seeking medical care from private clinics	524 (56.0)	278 (56.5)	246 (55.5)	226 (58.9)	298 (54.1)
Baseline prevalence, No. (%)^f^					
*S. mansoni*	397 (42.5)	195 (39.6)	202 (45.6)	165 (43.0)	232 (42.1)
Hookworm	372 (39.8)	175 (35.6)	197 (44.5)	130 (33.9)	242 (43.9)

Abbreviations: ALB, albendazole; EPG, eggs per gram. PZQ, praziquantel; SD, standard deviation.

^a^ The number of homes in the 17 study villages ranged from 68 to 411 households with a mean (SD) of 215.12 (89.88) homes. Study participants had lived in their village a mean of 16.74 years, and approximately 47% did not belong to the majority tribe (Mudama or Musoga). Homes were most often made of mud walls and thatched roofs. Only 29.41% of the sample was Muslim; the rest identified as Christian.

^b^ Baseline EPG is the infection status before treatment distribution.

^c^ Educational level, the highest level attained, was an ordinal variable from 0–16. The levels were no education 0, primary 1–7, senior 1–6 (levels 8–13), diploma (level 14), some university (level 15), and completed university (level 16).

^d^ Occupation represents income-earning work for each individual. The reference category is no income-earning occupation, which included adults and children who did not work, as well as housewives.

^e^ Home quality score is a count variable in which the roof, wall, and floor materials were ranked in quality from 1 to 4 and summed. Scores ≤3 indicated the worst possible home quality, which was the case for 24.92% of the sample (233 of 935 individuals). Among treated individuals only 18.09% of PZQ recipients (89 of 492) and 19.27% of ALB recipients (74 of 384) had the worst quality homes, compared with 32.51% (144 of 443) and 28.86% (159 of 551) for PZQ and ALB nonrecipients, respectively.

^f^ Of the *S. mansoni* and hookworm infections, 12.78% were coinfections.

### Statistical Analysis

The data were analyzed with Stata software, version 13.1 (StataCorp). To identify who did not receive PZQ or ALB, the 935 individuals from group A in Figure 1 were examined. Three-level, hierarchical logistic regressions were used [22]**.** Individuals were nested in 510 households, located in 17 villages. Socioeconomic factors and the total homes in each village were predictors of PZQ and ALB receipt. Baseline individual infection intensity and baseline village infection prevalence of *S. mansoni* and hookworm were covariates, respectively, for PZQ and ALB. Because IVM is coadministered with ALB, this variable was examined in the ALB model but dropped due to collinearity. There was insufficient evidence that the error terms of the PZQ and ALB models were correlated (χ^2^ = 3.20; *P* = .07), so separate regressions were used.

Intraclass correlation (ICC) coefficients are reported to describe the correlation of drug receipt within a household [23]. The crude global *R*^2^ was calculated as the square of the correlation between predicted and actual values of drug receipt [24]. The proportional reduction of error variance explained by the full model (conditional *R*^2^) was calculated as explained by Nakagawa and Schielzeth [25]. Furthermore, the area under the receiver operating characteristic curve was reported [22] and corrected for overfitting with 10- and 5-fold cross-validation [26]. An auxiliary analysis of treatment impact on infection prevalence is provided in Supplementary Text and Supplementary Tables 2–5.

### Ethics

This study was reviewed and approved by the Uganda National Council of Science and Technology, the Office of the President in Uganda, and the Cambridge University Human Biological Research Ethics Committee.

## RESULTS

### Proportion of Individuals Receiving Treatment

For individuals from group A in Figure 1, which was used in the drug receipt model, 37.86% (354 of 935) received both PZQ and ALB, 14.76% (138 of 935) received PZQ without ALB, and 3.21% (30 of 935) received ALB without PZQ. Therefore, a large proportion of study participants (44.17%, 413/935) received neither PZQ nor ALB. Table 1 presents the personal characteristics of untreated and treated individuals.

### Reasons for Not Receiving Treatment

Coverage reported by CMDs in the drug registers did not differ significantly for PZQ and was approximately 10% lower for ALB than the coverage measured from household surveys (Supplementary Table 6). Household heads explained why individuals in their home did not receive any drugs (including IVM). Reasons were available for 86.29% (302 of 350) of these individuals (group A, Figure 1). No drug availability was the most common response for being untreated (70.53%; 213 of 302). Additional explanations included a lack of health education (12.25%; 37 of 302), ineligibility (11.26%; 34 of 302), and noncompliance (6.0%; 18 of 302) (see Supplementary Text for detailed definitions of these reasons).

### Factors Describing Nonrecipients of PZQ

**Figure 2.**
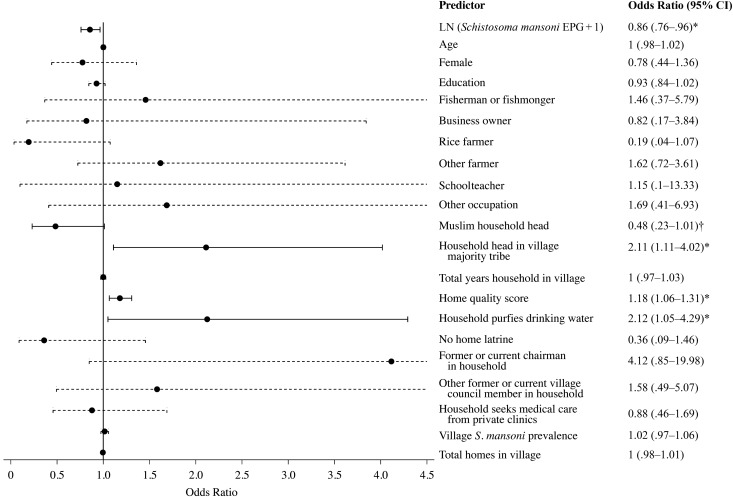
Determinants of praziquantel (PZQ) treatment receipt. The odds ratios are shown from the full multilevel model presented in Supplementary Table 7. The reference line at 1 indicates no increase or decrease in the likelihood of PZQ receipt. The 95% confidence intervals (CIs) also are plotted, and significant odds ratios are presented with solid lines. Occupation represents income-earning work for each individual; the reference category is no income-earning occupation, which included adults and children who did not work, as well as housewives. *P < .05; ^†^*P* = .054. Abbreviations: EPG, eggs per gram; LN, natural log.

Figure 2 presents the factors predicting PZQ treatment; the full model is presented in Supplementary Table 7. PZQ receipt was correlated between children and adults in the same household. Individuals within the same household were 76.35% more likely to have the same drug receipt status than individuals of different households in their village (ICC; Supplementary Table S7). For individual characteristics, only heavy *S. mansoni* infection intensity was associated with a decreased probability of PZQ treatment (*P* = .01).

Four household-level socioeconomic factors influenced PZQ distribution. Belonging to a household with a Muslim head was associated with a 51.63% lower likelihood of receiving PZQ than belonging to a household with a Christian head (this was borderline significant; *P* = .054). Muslims were the minority religion in the study area (Table 1). Compared with Christians, however, Muslims were neither less educated (mean educational level, 3.70 for Muslims and 3.36 for Christians; *P* = .12) nor more noncompliant (2.18% (6/275) of Muslims were noncompliant vs 2.00% (13/660) of Christians; *P* = .83). Moreover, there were Muslim CMDs (9 of 34) in the predominantly Muslim villages. Kinship was another social factor affecting PZQ distribution. Households in the village majority tribe were 2.11 times more likely to receive PZQ than households in minority tribes (*P* = .02). For household wealth, each point increase in home quality score (eg, improvement in wall materials from mud to aluminum or from aluminum to brick) increased the probability that the CMD would provide PZQ to an individual by 17.97% (*P* = .002). Households that purified drinking water were 2.12 times more likely to receive PZQ than households that did not treat, filter, or boil water collected from streams or lakes *P* = .04.

### Factors Describing Nonrecipients of ALB

**Figure 3.**
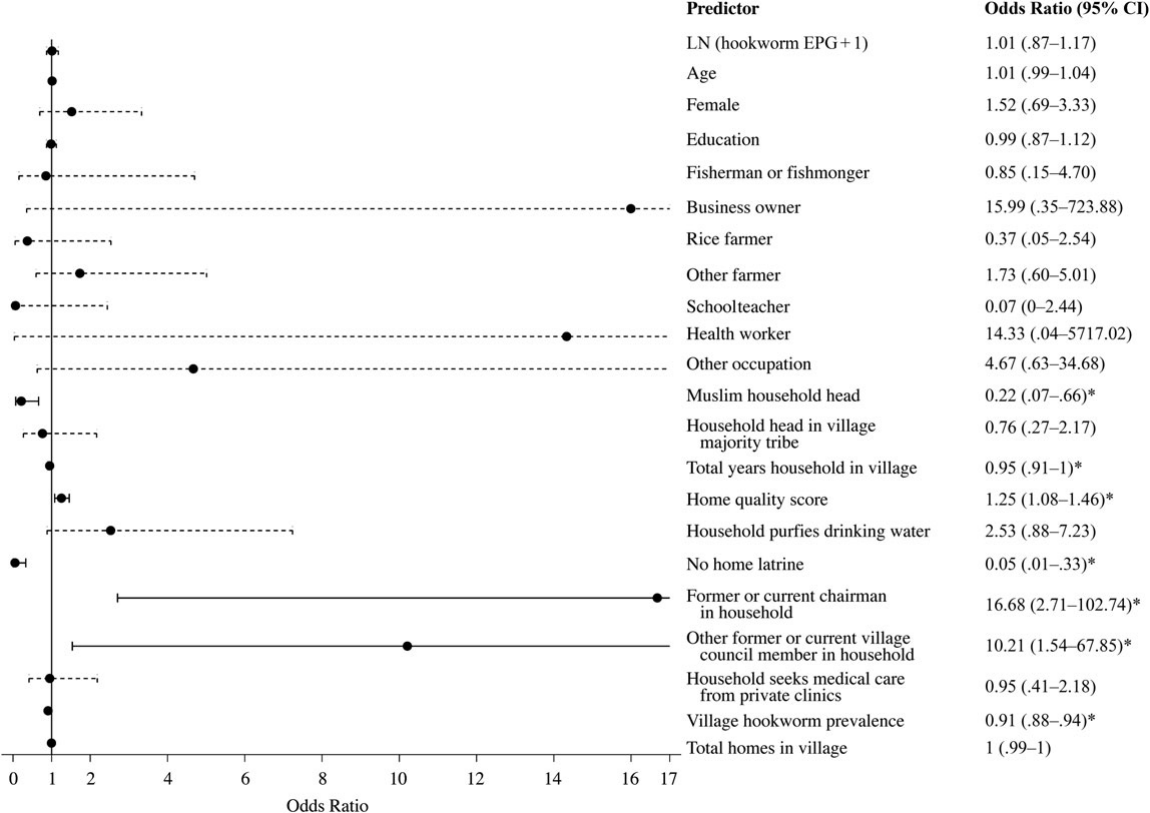
Determinants of albendazole (ALB) receipt. The odds ratios are shown from the full multilevel model presented in Supplementary Table 8. The reference line at 1 indicates no increase or decrease in the likelihood of ALB receipt. The 95% confidence intervals (CIs) are also plotted, and significant odds ratios are presented with solid lines. Occupation represents income-earning work for each individual; the reference category is no income-earning occupation, which included adults and children who did not work, as well as housewives. *P < .05. Abbreviations: EPG, eggs per gram; LN, natural log.

Figure 3 presents the determinants of ALB receipt; the full model is provided in Supplementary Table 8. ALB receipt was correlated between individuals within the same household. Children and their caretakers were 91.74% more likely to have the same ALB receipt status than individuals of different households in their village (ICC; Supplementary Table 8).

Individual-level variables were insignificant (*P* > .05) for ALB distribution. However, household-level socioeconomic factors were relevant for ALB receipt. Individuals in homes with Muslim household heads were 78.45% less likely to receive ALB than those in Christian households (*P* = .007). For each point increase in home quality score, the probability of ALB treatment increased by 25.40% (*P* = .003). In Uganda, the chairman has the highest formal position in the village government. Compared with households with no village government members, ALB receipt was 16.68 times more likely when the household included a former or current chairman (*P* = .002). Similarly, any current or former village government member in the household (not including chairmen) was associated with a 10.21-fold increase (*P* = .02) in the likelihood of ALB treatment. For each additional year that the household had been settled in their village, individuals within that home were 5% less likely to receive ALB (*P* = .03). The lack of access to a shared or private home latrine, which excludes public latrines, was related to a 94.80% lower likelihood of ALB receipt (*P* = .002).

One village-level factor affected ALB distribution. Individuals belonging to villages with high hookworm infection prevalence were 9.15% less likely to receive ALB than those in villages with low hookworm infection prevalence (*P* < .001). Determinants of ALB receipt remained robust when health workers were removed from the analysis (Supplementary Table 9). Moreover, all results for PZQ and ALB receipt remained when baseline *S. mansoni* and hookworm infection intensities were removed from the multilevel regressions (Supplementary Tables 10 and 11). For reference, unadjusted univariate regressions are shown in Supplementary Table 12.

## DISCUSSION

World Health Organization bulletins from 2013 and 2014 indicate that more than 200 and 600 million persons who require, respectively, PZQ for *S. mansoni* infections and ALB or mebendazole for hookworm infections do not receive treatment through MDA [6, 7]. We profiled untreated individuals in Mayuge District, Uganda, during the context of routine, community-based MDA. More than 44% of 935 individuals in 17 villages did not receive PZQ or ALB.

Existing World Health Organization guidelines prioritize individuals for treatment based on age, sex, and high-risk occupations for *S. mansoni* or hookworm infections [3], but these infection risk factors were unable to identify nonrecipients of MDA. Two household-level factors profiled untreated individuals: socioeconomic status and minority group affiliation. Schistosomiasis and hookworm infections are prevalent among the rural poor, who lack access to safe water and sanitation. We found that within such impoverished communities, socioeconomic divisions persist and determine who receives treatment for intestinal helminths. Individuals of low economic standing, who belonged to households with poor home quality, no access to purified water, and no home latrine, were less likely to receive PZQ or ALB. Similarly, low social status was negatively associated with treatment receipt. Households without members in the current or former village government were >10 times less likely to be treated. Belonging to a minority religion or tribe was also negatively associated with drug receipt.

Targeting observable socioeconomic factors could not only expand treatment coverage but also affect the prevalence of infection and address treatment fatigue. Nonrecipients had either heavy *S. mansoni* infection intensities or belonged to villages with a high prevalence of hookworm infection. Long-term residents were also 5% less likely to receive ALB, though this was a small effect. These individuals might experience treatment fatigue because of previous participation in MDA, which has been ongoing in the study area for 10 years [27]. To externally validate these profiles of untreated individuals, future studies are required in other countries implementing MDA.

Enough pills to treat all villagers were provided to CMDs and available in the village. However, 70% of untreated individuals believed drugs were not available because they had not been approached by CMDs and had not heard of the drug availability from other villagers. These untreated individuals indicated that they would comply if offered treatment by CMDs. Fleming et al [28] show in our study region that CMDs request remuneration for the time required to treat all households, but this does not explain why households of low socioeconomic status were disproportionately left untreated. Assuming that households of low socioeconomic status were hard to reach seems unlikely, because all individuals were found and contacted by CMDs in a baseline parasitology task before MDA (see Data Sources in the “Methods” section). To assess why households of lower socioeconomic status were not treated, research is needed on village social interactions [29]. Potential issues to explore include the influence of high social status households on CMDs, the similarity of treated households to CMDs, and the frequency of social interactions between CMDs and untreated households.

The analysis of coverage from MDA drug registers has been shown to be problematic owing to overreporting [30]. However, no significant differences were found between reported coverage from the drug registers and self-reported coverage from household surveys (Supplementary Table 6). This may be due to the sampling procedure used in this study, whereby study participants were selected in a task that was unrelated to MDA monitoring and was undertaken before the training and delivery of drugs to CMDs. The CMDs were unaware of future monitoring, surprise visits occurred, and no payments were provided for distribution. This method cannot increase the number of individuals who are recorded in drug registers by CMDs [11]. However, selecting individuals before administering and recording treatments may ensure the validity of CMD-reported coverage and facilitate monitoring of individuals who are not included in the national drug registers.

This study demonstrated how routinely collected drug registers can be used to obtain profiles of MDA nonrecipients. In Mayuge District, an area that has received repeated MDA treatments for *S. mansoni* and hookworm infections [27], coverage was dependent on household socioeconomic factors. Infection risk factors were not relevant for identifying untreated individuals during community-based distribution when compared with social characteristics, even though the study area had *S. mansoni* and hookworm infection prevalence of greater than 50% [31, 32]. Household socioeconomic factors identified the individuals failing to benefit from PZQ and ALB treatments and can be targeted to expand MDA coverage.

## Supplementary Data

Supplementary materials are available at http://cid.oxfordjournals.org. Consisting of data provided by the author to benefit the reader, the posted materials are not copyedited and are the sole responsibility of the author, so questions or comments should be addressed to the author.

Supplementary Data
