# Supplementary material for: Profiling Nonrecipients of Mass Drug Administration for Schistosomiasis and Hookworm Infections: A Comprehensive Analysis of Praziquantel and Albendazole Coverage in Community-Directed Treatment in Uganda
Source: Clin Infect Dis. 2015 Sep 25;62(2):200–7. doi: 10.1093/cid/civ829 (PMC4690482; doi:10.1093/cid/civ829)
Supplement: Supplementary Data [file supp_62_2_200__index.html]

Profiling non-recipients of mass drug administration for schistosomiasis and hookworm infections: a comprehensive analysis of praziquantel and albendazole coverage in community-directed treatment in Uganda — Profiling Nonrecipients of Mass Drug Administration for Schistosomiasis and Hookworm Infections: A Comprehensive Analysis of Praziquantel and Albendazole Coverage in Community-Directed Treatment in Uganda — Profiling Nonrecipients of Mass Drug Administration for Schistosomiasis and Hookworm Infections: A Comprehensive Analysis of Praziquantel and Albendazole Coverage in Community-Directed Treatment in Uganda — Supplementary Data 

# Profiling Nonrecipients of Mass Drug Administration for Schistosomiasis and Hookworm Infections: A Comprehensive Analysis of Praziquantel and Albendazole Coverage in Community-Directed Treatment in Uganda

## Supplementary Data

Supplementary Data

- Supplementary Data - Docx file
