# Supplementary material for: Profiling Nonrecipients of Mass Drug Administration for Schistosomiasis and Hookworm Infections: A Comprehensive Analysis of Praziquantel and Albendazole Coverage in Community-Directed Treatment in Uganda
Source: Clin Infect Dis. 2015 Sep 25;62(2):200–7. doi: 10.1093/cid/civ829 (PMC4690482; doi:10.1093/cid/civ829)
Supplement: Supplementary Data [file supp_civ829_civ829supp.docx]

**Supplementary information for** “Profiling non-recipients of mass drug administration for schistosomiasis and hookworm infections: a comprehensive analysis of praziquantel and albendazole coverage in community-directed treatment in Uganda”

Goylette F. Chami, Andreas A. Kontoleon, Erwin Bulte, Alan Fenwick, Narcis B. Kabatereine, Edridah M. Tukahebwa, David W. Dunne

Table of Contents

Text S1: Supplementary methods 2

Table S1: Differences between individuals included and not included in the household survey 4

Table S2: Descriptive statistics for follow-up/cohort sample 5

Table S3: Factors determining follow-up attrition 6

Table S4: Treatment effects model for PZQ impact on *S. mansoni* prevalence 7

Table S5: Treatment effects model for ALB impact on hookworm prevalence 9

Table S6: Paired t-tests of coverage in national registers and self-reported coverage 11

Table S7: Main model for determinants of praziquantel treatment receipt 12

Table S8: Main model for determinants of albendazole treatment receipt 13

Table S9: Main model for determinants of albendazole receipt without health workers 14

Table S10: Main model for determinants of praziquantel receipt without *S. mansoni* infection intensity 15

Table S11: Main model for determinants of albendazole receipt without hookworm infection intensity 16

Table S12: Univarate regressions for praziquantel and albendazole receipt 17

References 18

## Text S1: Supplementary methods

**Village selection**

This study was conducted from August-November 2013 in 17 villages of Mayuge District, Uganda. Five criteria were used to select and to ensure comparability of the study villages (unpublished data from VCD). These criteria include: 1) history of repeated MDA, 2) eligibility for PZQ treatment, 3) scheduled MDA in 2013, 4) documented *S. mansoni* or hookworm infection prevalence, and 5) similarity of village infrastructure. On a preliminary trip in February 2013, 41 villages were visited with the District Health Officer (DHO). These villages were located in eight of thirteen sub-counties in Mayuge District that received at least two consecutive years (2011-2012) of MDA. Although the full history of treatment is unavailable at the sub-county level, Mayuge District has received eight rounds of PZQ distribution from 2003-2012 and at least six rounds of ALB administration from 2006-2012. All villages were within five kilometers of Lake Victoria, which was guaranteed PZQ treatments from the VCD due to known *S. mansoni* transmission [1]. Village waypoints were recorded and available infrastructure such as public taps, latrines, and government facilities were surveyed with village chairmen. Using these local maps, 11 villages were dropped that differed in development from the majority of sampled villages. These villages were dropped because they were established towns with large trading centers, within one kilometer of a government hospital, or surrounded by government forests and isolated from other villages. From the remaining 30 villages, 17 villages were selected where national mapping in these villages or nearby primary schools showed greater than 30% prevalence of *S. mansoni* or hookworm infection.

**Participant selection**

Individuals were selected by gender and household position. Equal numbers of fathers with daughters, fathers with sons, mothers with daughters, and mothers with sons were requested. These divisions also facilitated the recruitment of adult males to achieve gender balance in the sample. Children and adults were further stratified to capture high-risk groups for *S. mansoni* or hookworm infections. The children sought were at least five years old with a target age between 10-14 years. Five years is the minimum age that is eligible for PZQ treatments and *S. mansoni* infection intensity peaks between 10-14 years in most *S. mansoni* endemic areas [2]. For adults, CMDs were asked to include fisherman and farmers, which are occupations with frequent exposure to *S. mansoni* [3, 4] and hookworm[5], respectively.

**Definitions of socio-economic variables**

The following socio-economic factors were measured from the household survey. For each individual, age (in years), the highest level of education completed, gender, and income-earning occupation (if any) were included. Education was a count variable of the highest level of education attained for that individual. Education was marked in levels from zero (no education) to 16. The levels of education were primary 1-7, senior 1-6 (levels 8-13), diploma (level 14), some university (level 15), and completed university (level 16). Occupation was defined as the income-earning occupation of the individual and was represented as a categorical variable. The base/reference category for occupation was ‘no income earning occupation,’ which included adults and children who did not work and housewives. The household level predictors are as follows. Binary indicators were used for the religion of the household head and the belonging of the household to the majority tribe (Mudama or Musoga in the study area) of their village. In the study area, wives may be from different tribes. However, the children take the tribe of the father (most always the household head). The measurement of water purification by the household is a dummy indicator and is positive if a household does any of the following activities to make their water safer to drink: boil, add bleach, strain through cloth, use water filter, solar disinfect, wash jerry can with soap, or let stand and settle. ‘No home latrine’ indicates that the household has no facilities and engages in open defecation in a bush or field. Two binary variables were used to measure household involvement in all village government positions, which is called the Local Council in Uganda. ‘Chairman’ is positive if any adult in the home has ever been the chairman of their village. Another binary indicator was constructed for the other local council members and is positive if any adult in the home has ever been vice chairman, secretary, defense, gender secretary, disabled secretary, youth council, elderly secretary, or information secretary. ‘Private clinic users’ also is a binary indicator of households that said they frequently seek medical supplies and care from drug shops and private health clinics. ‘Years in village’ is the total years that the household has settled in the village. ‘Home quality score’ is a count variable representing the total score of floor, walls, and roof materials. The quality of the roof, wall, and floor were ranked from 1-4 and summed. The rank order of the materials was grass, sticks, plastic, and metal for the roof; mud and sticks, plastic, metal, and bricks or cement for the walls; mud, plastic, wood planks, and brick or cement for the floor. Mud included cow dung. If no roof, wall, or floor material was present then zero was recorded. Additionally, the total number of homes (village size) was counted and included in the analyses.

**Definitions of reasons for not receiving drugs**

The lack of drug availability is defined as one of the following responses: “did not know”, “forgot”, “not available”, “not knowing where to receive drugs”, “only available for children”, “was outside the village”, and “was in the village, but away from home”. Ineligibility is defined as the household head indicating that the individual was ineligible for treatment due to age or extreme sickness. The lack of drug education includes the following responses “did not know the purpose of drugs”, “does not know the benefits of the drug”, “drugs don’t work”, “had to pay for drugs”, “no symptoms”, and “witchcraft causes infection”. Noncompliance was the refusal to take drugs and the following reasons were given by the respondents “bad side effects”, “clan differences with the drug distributor”, “tribe differences with the distributor”, “receiving the drugs and giving them to animals”, “friends don’t take drugs”, “neighbours don’t take drugs”, and “having no food or drink available with the drugs”. In the household survey, an option of ‘other’ was included and explained as an option for individuals who preferred not to provide a reason for not taking drugs or respondents that did not know why a person in their household took drugs. This category may have included other explanations for not receiving drugs, but, qualitatively, this option was rarely used.

**Statistical analysis for supplementary models of mass drug administration impact**

This section describes the methods used to analyze the impact of praziquantel (PZQ) and albendazole (ALB) receipt, respectively, on *S. mansoni* and hookworm infection prevalence (Supplementary Tables S3-S5). There was insufficient evidence from the in-text drug receipt models to suggest that the only difference between untreated and treated individuals was their treatment status, i.e. drugs were not distributed randomly across the covariates. To account for these selection biases, treatment effect models with doubly robust estimators were used to analyze mass drug administration (MDA) impact. Treatment effect models measured the MDA impact on participants when compared to no available MDA or an alternative program. However, treatment effect models cannot produce unknowable counterfactuals so that the only difference between MDA participants and non-participants is the actual receipt of treatment. Although separate treatment effect models were constructed for *S. mansoni* and hookworm prevalence, the specifications were similar.

Observations from 779 individuals in Group B (Figure 1, main text) were used and inverse-probability-weighted-regression adjustments were employed as described in Cattaneo 2010 [6] and Emsley et al. 2008 [7]. Robust standard errors also were used [8]. Doubly robust methods simultaneously estimate equations for the treatment receipt and outcome. This approach allows one of these equations to be misspecified, but the treatment effects model remains consistent. The treatment model was a logit regression with the same specifications as the drug receipt model for PZQ and ALB that is described in the main text. Binary variables indicating if an individual had at least one detectable egg per gram (EPG) of *S. mansoni* or hookworm after MDA were used as the dependent variables in the outcome models. The outcome model also was specified with the same covariates as the drug receipt (treatment) model. Health workers were excluded from both the treatment and outcome models for *S. mansoni* follow-up infection. This variable was excluded, as there was no variation in praziquantel receipt; all health workers received praziquantel. For the treatment effects model measuring MDA impact on infection prevalence, a potential outcomes framework was used where the potential outcomes (post-MDA infection) with and without treatment were estimated for every individual. The average treatment effect was calculated as the difference in the potential outcome means (expected post-MDA prevalence) for being treated versus untreated for all 779 individuals.

There are limitations to the analysis of prevalence reduction from MDA. In areas of high transmission, assessing the ability of MDA to cure infected individuals or maintain zero EPG for uninfected individuals is an overly strict measure of impact. Cure rates or maintaining no infection favors less infected individuals who have few EPG and infrequent exposure to parasites and, in turn, lower likelihood of reinfection. However, we present these models to enable comparisons to studies conducted during MDA.

## Table S1: Differences between individuals included and not included in the household survey

| **A) Baseline sample** | | **Individual lost to attrition due to lack of household survey information** | | |  | |  | |  | |  |
| --- | --- | --- | --- | --- | --- | --- | --- | --- | --- | --- | --- |
|  |  |  |  |  |  | |  | |  | |  |
| **Variable** | **Statistic** | **no** | **yes** | | **t** | | **p-value** | | **degrees of freedom** | |  |
| Baseline *S. mansoni* EPG | mean | 219.696 | 182.788 | | 0.416 | | 0.678**^a^** | | 1032 | |  |
|  | std. err. | 28.465 | 45.611 | |  | |  | |  | |  |
|  | obs. | 935 | 99 | |  | |  | |  | |  |
| Baseline hookworm EPG | mean | 348.578 | 120.121 | | 1.525 | | 0.128**^b^** | | 1032 | |  |
|  | std. err. | 48.603 | 31.534 | |  | |  | |  | |  |
|  | obs. | 935 | 99 | |  | |  | |  | |  |
| Age | mean | 24.179 | 22.081 | | 1.191 | | 0.234 | | 1032 | |  |
|  | std. err. | 0.541 | 1.801 | |  | |  | |  | |  |
|  | obs. | 935 | 99 | |  | |  | |  | |  |
| Female | frequency | 60.32% (564/935) | 59.60% (59/99) | | | |  | |  | |  |
|  | Chi-squared= 0.0196, p-value=0.889 | | |  | |  | |  | |  | |
|  |  |  |  | |  | |  | |  | |  |
| **B) Follow-up sample** | |  |  | |  | |  | |  | |  |
|  |  | **Individual lost to attrition due to lack of household survey information** | | |  | |  | |  | |  |
| **Variable** | **Statistic** | **no** | **yes** | | **t** | | **p-value** | | **degrees of freedom** | |  |
| Baseline *S. mansoni* EPG | mean | 190.444 | 205.185 | | -0.172 | | 0.863**^a^** | | 858 | |  |
|  | std. err. | 27.090 | 55.010 | |  | |  | |  | |  |
|  | obs. | 779 | 81 | |  | |  | |  | |  |
| Baseline hookworm EPG | mean | 358.721 | 110.074 | | 1.470 | | 0.142**^b^** | | 858 | |  |
|  | std. err. | 54.413 | 34.096 | |  | |  | |  | |  |
|  | obs. | 779 | 81 | |  | |  | |  | |  |
| Age | mean | 24.150 | 21.877 | | 1.158 | | 0.247 | | 858 | |  |
|  | std. err. | 0.597 | 2.030 | |  | |  | |  | |  |
|  | obs. | 779 | 81 | |  | |  | |  | |  |
| Female Chi-squared test | frequency | 61.10% (476/779) | 56.79% (46/81) | | | |  | |  | |  |
|  | Chi-squared = 0.5724, p-value=0.449 | | |  | |  | |  | |  | |
| EPG is an abbreviation for eggs per gram. | | | | | |  | |  | |  | |
| **^a^**P-values remain greater than 0.10 when Natural Log(*S. mansoni* eggs per gram+1) was used. | | | | | | | | | | | |
| **^b^**P-values remain greater than 0.10 when Natural Log(Hookworm eggs per gram +1) was used. | | | | | | | | | | | |

Panel A of Table S1 presents the two sample t-tests for individuals that were not included in the 935 individuals analyzed for the drug receipt models. The column indicating that individuals were lost to attrition represents the sample of 99 individuals that were in the initial baseline sample (1,034 individuals), but not included in the study (main analyses in-text), as they were not interviewed for the household questionnaire. Only the variables of baseline infection intensity, age, and gender were available for the baseline participants that were not surveyed in the household questionnaire. Chi-squared tests are provided for the binary variable of gender. Panel B presents the same analysis, but applied to the follow-up participants, which were used in the supplementary analysis of mass drug administration impact. There were no differences in age, baseline infection intensity, and gender between the 81 individuals that had follow-up parasitology but no household survey data, and the 779 individuals with follow-up parasitology and household survey data.

## Table S2: Descriptive statistics for follow-up/cohort sample

|  | **Follow-up sample** | |
| --- | --- | --- |
| **Variables** | *Mean* [Total] | *Std. Dev.* [%] |
| Baseline *S. mansoni* EPG | 190.444 | 756.086 |
| Baseline Hookworm EPG | 358.721 | 1518.705 |
| Age | 24.150 | 16.668 |
| Female | 476 | 0.611 |
| Education | 3.372 | 2.927 |
| No income-earning occupation | 506 | 0.650 |
| Fisherman or fishmonger | 33 | 0.042 |
| Business owner | 20 | 0.026 |
| Rice farmer | 28 | 0.036 |
| Other farmer | 152 | 0.195 |
| School teacher | 9 | 0.012 |
| Health worker | 3 | 0.004 |
| Other occupation | 28 | 0.036 |
| Muslim household head | 214 | 0.275 |
| Household head belongs to village majority tribe | 418 | 0.537 |
| Total years household settled in village | 16.606 | 11.441 |
| Home quality score | 7.372 | 3.245 |
| Household purifies drinking water | 317 | 0.407 |
| No home latrine | 46 | 0.059 |
| Former or current village chairman in household | 37 | 0.047 |
| Other former or current village government member in household | 67 | 0.086 |
| Household seeks medical care from private clinics | 445 | 0.571 |
| Village baseline *S. mansoni* prevalence | 323 | 0.415 |
| Village baseline hookworm prevalence | 315 | 0.404 |
| **Observations** | 779 |  |

Table S2 provides the summary statistics of the cohort analyzed (779 individuals) for the supplementary analyses of mass drug administration impact (Group B of Figure 1 in the main text). EPG is an abbreviation for eggs per gram. Baseline EPG is the infection status before treatment distribution. Occupation represents income-earning work for each individual. The reference category for occupation is ‘no income-earning occupation’. This category included adults and children who did not work as well as housewives.

## Table S3: Factors determining follow-up attrition

|  | **Random intercept model^a^** | | | | |
| --- | --- | --- | --- | --- | --- |
| **Variable** | **Estimate^b^** | **Std. err.** | **p-value** | **95% CI** | |
| **Fixed component** |  |  |  |  |  |
| Baseline LN (*S. mansoni* EPG +1) **^c^** | 0.916 | 0.066 | 0.224 | 0.796 | 1.055 |
| Baseline LN (Hookworm EPG +1) **^c^** | 1.055 | 0.076 | 0.456 | 0.917 | 1.214 |
| Age | 0.992 | 0.013 | 0.553 | 0.967 | 1.018 |
| Female | 1.200 | 0.462 | 0.635 | 0.565 | 2.551 |
| Education | 0.877 | 0.054 | 0.033 | 0.778 | 0.989 |
| Fisherman or fishmonger**^d^** | 2.131 | 1.811 | 0.373 | 0.403 | 11.275 |
| Business owner**^d^** | 2.623 | 2.507 | 0.313 | 0.403 | 17.082 |
| Rice farmer**^d^** | 0.574 | 0.478 | 0.505 | 0.112 | 2.941 |
| Other farmer**^d^** | 2.099 | 1.222 | 0.203 | 0.671 | 6.568 |
| School teacher**^d^** | 16.952 | 50.451 | 0.342 | 0.050 | 5788.213 |
| Health worker**^d^** | 0.016 | 0.027 | 0.016 | 0.001 | 0.464 |
| Other occupation**^d^** | 0.952 | 0.756 | 0.951 | 0.201 | 4.510 |
| Muslim household head | 0.227 | 0.140 | 0.016 | 0.068 | 0.761 |
| Household head belongs to village majority tribe | 1.402 | 0.572 | 0.408 | 0.630 | 3.119 |
| Total years household settled in village | 0.984 | 0.020 | 0.429 | 0.945 | 1.024 |
| Home quality score | 1.015 | 0.069 | 0.824 | 0.888 | 1.160 |
| Household purifies drinking water | 0.695 | 0.295 | 0.392 | 0.303 | 1.598 |
| No home latrine | 1.077 | 0.908 | 0.930 | 0.207 | 5.618 |
| Former or current village chairman in household | 2.167 | 3.999 | 0.675 | 0.058 | 80.619 |
| Other former or current village government member in household | 1.024 | 0.754 | 0.974 | 0.242 | 4.339 |
| Household seeks medical care from private clinics | 1.123 | 0.456 | 0.775 | 0.507 | 2.488 |
| Constant | 165.539 | 197.859 | <0.001 | 15.904 | 1723.024 |
| **Random component** |  | | |  |  |
| Household | 13.857 | 5.675 |  | 6.209 | 30.923 |
| **Intraclass correlation** |  |  |  |  |  |
| Household | 0.808 | 0.064 |  | 0.654 | 0.904 |
| Obs. 935 |  |  |  |  |  |
| **ROC area** | 0.993 | 0.002 |  | 0.989 | 0.996 |
| **Crude Global R^2^** | 0.636 |  |  |  |  |
| **Conditional R^2^** | 0.809 |  |  |  |  |

**^a^** A two level hierarchical model with 510 observations at level two and 935 observations at level one is presented with a binomial family and a logit link. Random effects are presented to allow for variance at the household level.

**^b^** For the fixed and random effects portions of the model, respectively, the estimate represents the odds ratios and variance.

**^c^** LN and EPG are abbreviations for natural log and eggs per gram, respectively.

**^d^** Occupation represents income-earning work for each individual. The reference category for occupation is ‘no income-earning occupation’. This category included adults and children who did not work as well as housewives. The global p-value equals 0.184 for all occupations.

Prior to analyzing the impact of MDA on infection prevalence or intensity, an attrition model was constructed to assess the loss of individuals from Group A to Group B of Figure 1 (main text). The dependent variable in this logit model is a binary indicator representing if someone was included in the follow-up sample (779 people from Group B of Figure 1 in the main text). This follow-up sample was used to analyze treatment impacts. Village-level variables were excluded from this model, as the households were already selected during baseline. Individual or household level factors were the only characteristics that could be selected upon at this time. Here, we examined what factors may differ between the group used to assess drug receipt and the group used to assess treatment impact. The only variable that differs in this attrition model and is significant for drug receipt of PZQ or ALB (Tables S7-S8) is religion. It appears that individuals with a Muslim household head were less likely to return or be brought back by the community medicine distributor (CMDs) for a follow-up stool sample after mass drug administration. These individuals were also less likely to receive praziquantel (PZQ) or albendazole (ALB) (Tables S7-S8) from CMDs. Education and being a health worker also were significant for predicting attrition, but both were insignificant in the PZQ or ALB drug receipt models (Tables S7-S8).

## Table S4: Treatment effects model for PZQ impact on *S. mansoni* prevalence

|  | **Infection prevalence after MDA** | **Coef.** | **Robust Std. err.** | **p-value** | **95% Confidence interval** | | |
| --- | --- | --- | --- | --- | --- | --- | --- |
| **Positive *S. mansoni* infection (at least one EPG) after MDA** | Difference between *S. mansoni* prevalence with PZQ and without PZQ (ATE) | -0.125 | 0.027 | <0.001 | -0.179 | -0.071 | |
|  | *S. mansoni* prevalence without PZQ (Potential outcome with no PZQ receipt) | 0.405 | 0.023 | <0.001 | 0.360 | 0.451 | |
|  |  |  |  |  |  |  | |
|  | **Variables** | **Coef.** | **Robust Std. err.** | **p-value** | **95% Confidence interval** | | |
| **Potential outcome model (Logit) for PZQ=1** | Baseline LN(*S. mansoni* EPG + 1) ^a^ | 0.442 | 0.075 | <0.001 | 0.295 | | 0.589 |
|  | Age | -0.021 | 0.014 | 0.143 | -0.049 | | 0.007 |
|  | Female | 0.069 | 0.329 | 0.834 | -0.575 | | 0.714 |
|  | Education | 0.026 | 0.065 | 0.686 | -0.101 | | 0.153 |
|  | Fisherman or fishmonger ^b^ | 1.117 | 0.951 | 0.240 | -0.746 | | 2.980 |
|  | Business owner ^b^ | 0.292 | 0.882 | 0.741 | -1.437 | | 2.022 |
|  | Rice farmer ^b^ | 0.214 | 0.699 | 0.759 | -1.156 | | 1.585 |
|  | Other farmer ^b^ | 0.416 | 0.548 | 0.448 | -0.659 | | 1.490 |
|  | School teacher ^b^ | 0.809 | 1.413 | 0.567 | -1.961 | | 3.578 |
|  | Health worker ^c^ | - | - | - | - | | - |
|  | Other occupation ^b^ | 0.992 | 0.592 | 0.093 | -0.167 | | 2.152 |
|  | Muslim household head | -0.308 | 0.331 | 0.352 | -0.956 | | 0.341 |
|  | Household head belongs to village majority tribe | -0.117 | 0.313 | 0.709 | -0.729 | | 0.496 |
|  | Total years household settled in village | 0.016 | 0.012 | 0.163 | -0.007 | | 0.040 |
|  | Home quality score | 0.001 | 0.050 | 0.979 | -0.096 | | 0.099 |
|  | Household purifies drinking water | 0.086 | 0.340 | 0.802 | -0.582 | | 0.753 |
|  | No home latrine | -0.027 | 0.466 | 0.953 | -0.940 | | 0.885 |
|  | Former or current village chairman in household | 0.356 | 0.582 | 0.541 | -0.784 | | 1.496 |
|  | Other former or current village government member in household | 0.106 | 0.487 | 0.828 | -0.848 | | 1.060 |
|  | Household seeks medical care from private clinics | -0.267 | 0.332 | 0.421 | -0.918 | | 0.384 |
|  | Village baseline *S. mansoni* prevalence | 0.049 | 0.010 | <0.001 | 0.029 | | 0.068 |
|  | Total homes in village | 0.001 | 0.002 | 0.729 | -0.004 | | 0.005 |
|  | Constant | -3.125 | 0.772 | <0.001 | -4.639 | | -1.611 |
| **Potential outcome model (Logit) for PZQ=0** | Baseline LN(*S. mansoni* EPG + 1) | 0.325 | 0.065 | <0.001 | 0.197 | | 0.453 |
|  | Age | -0.062 | 0.015 | <0.001 | -0.091 | | -0.032 |
|  | Female | -0.586 | 0.316 | 0.063 | -1.206 | | 0.033 |
|  | Education | 0.015 | 0.061 | 0.804 | -0.104 | | 0.134 |
|  | Fisherman or fishmonger | 0.881 | 0.861 | 0.306 | -0.807 | | 2.570 |
|  | Business owner | 0.851 | 1.357 | 0.531 | -1.808 | | 3.509 |
|  | Rice farmer | 3.131 | 0.992 | 0.002 | 1.187 | | 5.075 |
|  | Other farmer | 0.702 | 0.591 | 0.235 | -0.457 | | 1.860 |
|  | School teacher | -4.414 | 0.944 | <0.001 | -6.264 | | -2.563 |
|  | Health worker | - | - | - | - | | - |
|  | Other occupation | 0.693 | 0.787 | 0.378 | -0.849 | | 2.235 |
|  | Muslim household head | 0.033 | 0.392 | 0.932 | -0.735 | | 0.802 |
|  | Household head belongs to village majority tribe | -0.686 | 0.297 | 0.021 | -1.269 | | -0.104 |
|  | Total years household settled in village | 0.036 | 0.016 | 0.025 | 0.005 | | 0.067 |
|  | Home quality score | -0.074 | 0.045 | 0.102 | -0.162 | | 0.015 |
|  | Household purifies drinking water | -0.007 | 0.297 | 0.982 | -0.590 | | 0.576 |
|  | No home latrine | -0.794 | 0.585 | 0.174 | -1.940 | | 0.352 |
|  | Former or current village chairman in household | 0.444 | 0.666 | 0.505 | -0.861 | | 1.749 |
|  | Other former or current village government member in household | 0.070 | 0.445 | 0.874 | -0.802 | | 0.943 |
|  | Household seeks medical care from private clinics | 0.446 | 0.317 | 0.159 | -0.175 | | 1.067 |
|  | Village baseline *S. mansoni* prevalence | 0.045 | 0.008 | <0.001 | 0.028 | | 0.061 |
|  | Total homes in village | 0.001 | 0.002 | 0.559 | -0.002 | | 0.004 |
|  | Constant | -2.427 | 0.663 | <0.001 | -3.727 | | -1.127 |
| **Treatment model (Logit)** | Baseline LN(*S. mansoni* EPG + 1) | -0.056 | 0.036 | 0.125 | -0.126 | | 0.015 |
|  | Age | 0.004 | 0.006 | 0.470 | -0.007 | | 0.016 |
| **Dependent variable: PZQ receipt** | Female | -0.119 | 0.167 | 0.477 | -0.447 | | 0.209 |
|  | Education | 0.001 | 0.030 | 0.966 | -0.057 | | 0.059 |
|  | Fisherman or fishmonger | -0.063 | 0.391 | 0.872 | -0.829 | | 0.703 |
|  | Business owner | -0.777 | 0.554 | 0.161 | -1.864 | | 0.309 |
|  | Rice farmer | -2.040 | 0.505 | <0.001 | -3.028 | | -1.051 |
|  | Other farmer | 0.020 | 0.235 | 0.933 | -0.441 | | 0.481 |
|  | School teacher | -0.515 | 0.756 | 0.496 | -1.996 | | 0.967 |
|  | Health worker | - | - | - | - | | - |
|  | Other occupation | 0.628 | 0.456 | 0.168 | -0.265 | | 1.521 |
|  | Muslim household head | -0.583 | 0.174 | 0.001 | -0.923 | | -0.242 |
|  | Household head belongs to village majority tribe | 0.529 | 0.157 | 0.001 | 0.221 | | 0.836 |
|  | Total years household settled in village | -0.003 | 0.007 | 0.653 | -0.017 | | 0.011 |
|  | Home quality score | 0.102 | 0.025 | <0.001 | 0.053 | | 0.151 |
|  | Household purifies drinking water | 0.206 | 0.164 | 0.209 | -0.115 | | 0.528 |
|  | No home latrine | -0.152 | 0.340 | 0.655 | -0.818 | | 0.515 |
|  | Former or current village chairman in household | 0.611 | 0.396 | 0.123 | -0.166 | | 1.388 |
|  | Other former or current village government member in household | 0.224 | 0.282 | 0.426 | -0.328 | | 0.777 |
|  | Household seeks medical care from private clinics | -0.145 | 0.160 | 0.365 | -0.459 | | 0.169 |
|  | Village baseline *S. mansoni* prevalence | 0.008 | 0.004 | 0.060 | <0.001 | | 0.017 |
|  | Total homes in village | -0.002 | 0.001 | 0.051 | -0.004 | | <0.001 |
|  | Constant | -0.320 | 0.351 | 0.363 | -1.008 | | 0.368 |
|  | Obs. 776 ^c^ |  |  |  |  | |  |

^a^ LN and EPG are abbreviations for natural log and eggs per gram, respectively.

^b^ Occupation represents income-earning work for each individual. The reference category for occupation is ‘no income-earning occupation’. This category included adults and children who did not work as well as housewives.

^c^ Health workers (three individuals) were omitted as there was no variation in drug receipt amongst these individuals; all received PZQ.

The methods and specification of the model in Table S4 are described in the supplementary methods (Text S1). In brief, this model presents a potential outcomes framework to assess the change in *S. mansoni* prevalence attributable to receiving praziquantel (PZQ). The model employs a doubly robust approach to estimate outcome equations (Panels B-C) and a treatment receipt equation (Panel D). Panel A shows the expected *S. mansoni* prevalence in the context of no PZQ treatments and the change in that prevalence (average treatment effect (ATE)) with PZQ receipt. Panel B presents the effects of the covariates on the dependent variable of follow-up *S. mansoni* infection. This dependent variable is a binary variable indicating if someone was infected after mass drug administration (MDA) with at least one detectable egg per gram (EPG) in his or her stool sample. This regression considers the potential outcome (infection prevalence) from no one receiving PZQ. Panel C is setup in the same manner as Panel B, but considers the potential outcome (infection prevalence) from everyone receiving PZQ. Panel D is a model for treatment that is run simultaneously with the equations for Panels B & C. The dependent variable in this model is the actual receipt of PZQ. PZQ receipt decreased *S. mansoni* prevalence by 12.50% (p-value<0.001) from the prevalence of 40.54% (p-value<0.001) expected after MDA in an untreated population (Panel A).

## Table S5: Treatment effects model for ALB impact on hookworm prevalence

|  | **Infection prevalence after MDA** | **Coef.** | **Robust Std. err.** | **p-value** | **95% Confidence interval** | |
| --- | --- | --- | --- | --- | --- | --- |
| **Positive hookworm infection (at least one EPG) after MDA** | Difference between hookworm prevalence with ALB and without ALB (ATE) | -0.144 | 0.028 | <0.001 | -0.199 | -0.089 |
|  | Hookworm prevalence without ALB (Potential outcome with no ALB receipt) | 0.317 | 0.020 | <0.001 | 0.277 | 0.357 |
|  |  |  |  |  |  |  |
|  | **Variables** | **Coef.** | **Robust Std. err.** | **p-value** | **95% Confidence interval** | |
| **Potential outcome model (Logit) for ALB=1** | Baseline LN(Hookworm EPG + 1) ^a^ | 0.405 | 0.052 | <0.001 | 0.302 | 0.508 |
|  | Age | -0.002 | 0.010 | 0.836 | -0.021 | 0.017 |
|  | Female | -0.310 | 0.270 | 0.252 | -0.839 | 0.220 |
|  | Education | -0.022 | 0.056 | 0.698 | -0.131 | 0.088 |
|  | Fisherman or fishmonger ^b^ | -1.845 | 0.976 | 0.059 | -3.758 | 0.067 |
|  | Business owner ^b^ | -0.996 | 0.932 | 0.285 | -2.822 | 0.830 |
|  | Rice farmer ^b^ | 2.023 | 0.602 | 0.001 | 0.843 | 3.203 |
|  | Other farmer ^b^ | 0.456 | 0.359 | 0.204 | -0.248 | 1.161 |
|  | School teacher ^b^ | -6.880 | 0.791 | <0.001 | -8.431 | -5.329 |
|  | Health worker ^b^ | 10.082 | 0.743 | <0.001 | 8.626 | 11.538 |
|  | Other occupation ^b^ | 0.525 | 0.746 | 0.482 | -0.938 | 1.988 |
|  | Muslim household head | 0.247 | 0.288 | 0.392 | -0.318 | 0.812 |
|  | Household head belongs to village majority tribe | -0.063 | 0.282 | 0.822 | -0.616 | 0.489 |
|  | Total years household settled in village | 0.003 | 0.013 | 0.826 | -0.023 | 0.028 |
|  | Home quality score | 0.023 | 0.047 | 0.627 | -0.069 | 0.114 |
|  | Household purifies drinking water | 0.052 | 0.266 | 0.846 | -0.470 | 0.574 |
|  | No home latrine | 0.703 | 0.524 | 0.180 | -0.324 | 1.730 |
|  | Former or current village chairman in household | -0.698 | 0.564 | 0.216 | -1.804 | 0.408 |
|  | Other former or current village government member in household | 0.142 | 0.444 | 0.749 | -0.728 | 1.011 |
|  | Household seeks medical care from private clinics | -0.361 | 0.267 | 0.177 | -0.884 | 0.163 |
|  | Village baseline hookworm prevalence | 0.020 | 0.008 | 0.012 | 0.004 | 0.035 |
|  | Total homes in village | -0.003 | 0.002 | 0.130 | -0.006 | 0.001 |
|  | Constant | -2.001 | 0.821 | 0.015 | -3.610 | -0.392 |
| **Potential outcome model (Logit) for ALB=0** | Baseline LN(Hookworm EPG + 1) | 0.297 | 0.089 | 0.001 | 0.122 | 0.473 |
|  | Age | 0.024 | 0.012 | 0.040 | 0.001 | 0.047 |
|  | Female | 0.233 | 0.388 | 0.548 | -0.528 | 0.995 |
|  | Education | -0.020 | 0.062 | 0.746 | -0.141 | 0.101 |
|  | Fisherman or fishmonger | -0.814 | 0.840 | 0.333 | -2.460 | 0.832 |
|  | Business owner | -0.477 | 1.105 | 0.666 | -2.642 | 1.689 |
|  | Rice farmer | -1.925 | 1.373 | 0.161 | -4.616 | 0.767 |
|  | Other farmer | -0.888 | 0.556 | 0.110 | -1.978 | 0.202 |
|  | School teacher | 0.197 | 1.053 | 0.851 | -1.866 | 2.261 |
|  | Health worker | -6.754 | 0.751 | <0.001 | -8.226 | -5.282 |
|  | Other occupation | -1.274 | 0.886 | 0.151 | -3.011 | 0.463 |
|  | Muslim household head | 0.044 | 0.417 | 0.916 | -0.774 | 0.862 |
|  | Household head belongs to village majority tribe | 0.155 | 0.333 | 0.642 | -0.498 | 0.808 |
|  | Total years household settled in village | 0.010 | 0.015 | 0.510 | -0.019 | 0.039 |
|  | Home quality score | <0.001 | 0.050 | 0.996 | -0.099 | 0.098 |
|  | Household purifies drinking water | -0.079 | 0.365 | 0.829 | -0.795 | 0.637 |
|  | No home latrine | -1.189 | 1.130 | 0.292 | -3.403 | 1.024 |
|  | Former or current village chairman in household | 0.268 | 0.699 | 0.701 | -1.101 | 1.637 |
|  | Other former or current village government member in household | 0.261 | 0.512 | 0.611 | -0.742 | 1.263 |
|  | Household seeks medical care from private clinics | 0.445 | 0.381 | 0.242 | -0.301 | 1.192 |
|  | Village baseline hookworm prevalence | -0.015 | 0.011 | 0.184 | -0.037 | 0.007 |
|  | Total homes in village | -0.001 | 0.002 | 0.635 | -0.005 | 0.003 |
|  | Constant | -2.441 | 0.847 | 0.004 | -4.102 | -0.781 |
| **Treatment model (Logit)** | Baseline LN(hookworm EPG + 1) | 0.006 | 0.033 | 0.853 | -0.058 | 0.070 |
|  | Age | 0.008 | 0.006 | 0.196 | -0.004 | 0.019 |
| **Dependent variable: ALB receipt** | Female | 0.144 | 0.165 | 0.382 | -0.179 | 0.467 |
|  | Education | -0.037 | 0.030 | 0.226 | -0.096 | 0.023 |
|  | Fisherman or fishmonger | -0.206 | 0.410 | 0.615 | -1.010 | 0.598 |
|  | Business owner | -0.266 | 0.526 | 0.614 | -1.297 | 0.766 |
|  | Rice farmer | -0.890 | 0.459 | 0.053 | -1.790 | 0.010 |
|  | Other farmer | 0.016 | 0.234 | 0.945 | -0.442 | 0.474 |
|  | School teacher | -0.228 | 0.833 | 0.784 | -1.860 | 1.404 |
|  | Health worker | 0.036 | 1.164 | 0.975 | -2.245 | 2.318 |
|  | Other occupation | 0.660 | 0.465 | 0.156 | -0.251 | 1.571 |
|  | Muslim household head | -0.381 | 0.183 | 0.038 | -0.740 | -0.022 |
|  | Household head belongs to village majority tribe | 0.088 | 0.162 | 0.587 | -0.229 | 0.405 |
|  | Total years household settled in village | -0.025 | 0.007 | <0.001 | -0.039 | -0.011 |
|  | Home quality score | 0.089 | 0.026 | 0.001 | 0.038 | 0.139 |
|  | Household purifies drinking water | -0.010 | 0.166 | 0.951 | -0.336 | 0.316 |
|  | No home latrine | -0.350 | 0.362 | 0.333 | -1.059 | 0.359 |
|  | Former or current village chairman in household | 1.061 | 0.395 | 0.007 | 0.287 | 1.835 |
|  | Other former or current village government member in household | 0.407 | 0.276 | 0.140 | -0.134 | 0.949 |
|  | Household seeks medical care from private clinics | 0.048 | 0.163 | 0.769 | -0.272 | 0.367 |
|  | Village baseline hookworm prevalence | -0.025 | 0.005 | <0.001 | -0.034 | -0.016 |
|  | Total homes in village | -0.002 | 0.001 | 0.014 | -0.004 | <0.001 |
|  | Constant | 0.753 | 0.443 | 0.089 | -0.115 | 1.621 |
|  | Obs. 779 |  |  |  |  |  |

^a^ LN and EPG are abbreviations for natural log and eggs per gram, respectively.

^b^ Occupation represents income-earning work for each individual. The reference category for occupation is ‘no income-earning occupation’. This category included adults and children who did not work as well as housewives.

The methods used in the model in Table S5 are explained in the supplementary methods (Text S1) and briefly reiterated in the explanation of Table S4. Panel A presents the average treatment effect (ATE) from receiving albendazole (ALB) when compared to the potential prevalence outcome under no ALB treatment. The equations used to estimate the ATE are provided in Panels B-D. The dependent variable for Panels B and C was a binary indicator that was positive if an individual had hookworm infection (at least one detectable eggs per gram (EPG)) after mass drug administration (MDA). Panel B reports the potential outcome (prevalence after MDA) under the scenario that no one received ALB whereas Panel C reports the potential outcome considering that everyone received ALB. For Panel D, the dependent variable was a binary indicator of ALB receipt. The receipt of ALB reduced hookworm infection prevalence by 14.38% (p-value<0.001) when compared to the potential outcome of 31.68% (p-value<0.001) for a population that does not receive ALB (Panel A).

## Table S6: Paired t-tests of coverage in national registers and self-reported coverage

| **PZQ** |  | **Register-reported** | **Self-reported** | **t** | **p-value** | **df** |
| --- | --- | --- | --- | --- | --- | --- |
|  | mean | 0.523 | 0.537 | -0.742 | 0.459 | 914 |
|  | std. err. | 0.167 | 0.167 |  |  |  |
|  | Obs. | 903 | 903 |  |  |  |
| **ALB** | mean | 0.409 | 0.506 | -4.808 | <0.0001 | 902 |
|  | std. err. | 0.016 | 0.017 |  |  |  |
|  | Obs. | 915 | 915 |  |  |  |

For coverage reported in the national drug registers, 52.62% (492/935) of individuals received PZQ and 41.07% (384/935) of participants received ALB. As a robustness check, a paired t-test was used to compare this coverage that was reported by community medicine distributors (CMDs) with the self-reported coverage from the household surveys. Self-reported coverage is measured by the response of the household head. The household head was asked to indicate the drugs taken by other household members during the household survey. This measure may suffer from recall bias (failure to remember or simply not being present when other members received drugs) and is only provided as a crude validity check to the coverage reported in the national drug registers. There was no significant difference (53.71%, p-value=0.459) between the proportion of individuals reported as receiving PZQ in the national registers by CMDs and self-reported PZQ. However, almost 10% more individuals (50.60%, p-value<0.0001) said they received ALB than was recorded in the national drug registers by the CMDs.

## Table S7: Main model for determinants of praziquantel treatment receipt

|  | **A) Empty/variance components model**^a^ | | | | | **B) Random intercept model** ^a^ | | | | |
| --- | --- | --- | --- | --- | --- | --- | --- | --- | --- | --- |
| **Variable** | **Estimate**^b^ | **Std. err.** | **p-value** | **95% CI** | | **Estimate**^b^ | **Std. err.** | **p-value** | **95% CI** | |
| **Fixed component** |  |  |  |  |  | **Fixed component** | | |  |  |
| Baseline LN(*S. mansoni* EPG + 1) ^c^ |  |  |  |  |  | 0.856 | 0.052 | 0.011 | 0.759 | 0.965 |
| Age |  |  |  |  |  | 1.001 | 0.010 | 0.931 | 0.982 | 1.020 |
| Female |  |  |  |  |  | 0.775 | 0.222 | 0.375 | 0.442 | 1.359 |
| Education |  |  |  |  |  | 0.927 | 0.045 | 0.120 | 0.843 | 1.020 |
| Fisherman or fishmonger ^d^ |  |  |  |  |  | 1.458 | 1.026 | 0.592 | 0.367 | 5.789 |
| Business owner ^d^ |  |  |  |  |  | 0.818 | 0.646 | 0.799 | 0.174 | 3.843 |
| Rice farmer ^d^ |  |  |  |  |  | 0.194 | 0.169 | 0.060 | 0.035 | 1.075 |
| Other farmer ^d^ |  |  |  |  |  | 1.619 | 0.663 | 0.240 | 0.725 | 3.614 |
| School teacher ^d^ |  |  |  |  |  | 1.150 | 1.438 | 0.911 | 0.099 | 13.334 |
| Health worker ^e^ |  |  |  |  |  | - | - | - | - | - |
| Other occupation ^d^ |  |  |  |  |  | 1.686 | 1.217 | 0.469 | 0.410 | 6.934 |
| Muslim household head |  |  |  |  |  | 0.484 | 0.182 | 0.054 | 0.231 | 1.013 |
| Household head belongs to village majority tribe |  |  |  |  |  | 2.112 | 0.693 | 0.023 | 1.110 | 4.018 |
| Total years household settled in village |  |  |  |  |  | 1.000 | 0.014 | 0.991 | 0.973 | 1.028 |
| Home quality score |  |  |  |  |  | 1.180 | 0.062 | 0.002 | 1.065 | 1.307 |
| Household purifies drinking water |  |  |  |  |  | 2.124 | 0.762 | 0.036 | 1.051 | 4.292 |
| No home latrine |  |  |  |  |  | 0.361 | 0.257 | 0.152 | 0.089 | 1.456 |
| Former or current village chairman in household |  |  |  |  |  | 4.115 | 3.317 | 0.079 | 0.848 | 19.975 |
| Other former or current village government member in household |  |  |  |  |  | 1.582 | 0.940 | 0.440 | 0.494 | 5.068 |
| Household seeks medical care from private clinics |  |  |  |  |  | 0.878 | 0.293 | 0.697 | 0.457 | 1.688 |
| Village baseline *S. mansoni* prevalence |  |  |  |  |  | 1.015 | 0.021 | 0.474 | 0.974 | 1.058 |
| Total homes in village |  |  |  |  |  | 0.996 | 0.005 | 0.416 | 0.985 | 1.006 |
| Constant | 1.211 | 0.542 | 0.669 | 0.504 | 2.910 | 0.669 | 0.858 | 0.754 | 0.054 | 8.262 |
| **Random component** |  |  |  |  |  | **Random component** | | |  |  |
| Household | 8.747 | 2.340 |  | 5.178 | 14.776 | 7.815 | 2.162 |  | 4.544 | 13.441 |
| Village | 3.345 | 1.443 |  | 1.436 | 7.791 | 2.806 | 1.265 |  | 1.159 | 6.790 |
| **Intraclass correlation** |  |  |  |  |  | **Intraclass correlation** | | |  |  |
| Household | 0.786 | 0.045 |  | 0.684 | 0.862 | 0.764 | 0.050 |  | 0.652 | 0.847 |
| Village | 0.217 | 0.065 |  | 0.116 | 0.370 | 0.202 | 0.065 |  | 0.102 | 0.359 |
|  | Obs. 929 ^e^ |  |  |  |  | Obs. 929 ^e^ |  |  |  |  |
| **Likelihood ratio test of empty model vs. random intercept model** | | | | |  | **ROC area,** Obs. 929 | |  |  |  |
| Chi^2^(21) 52.08, p-value=0.0002 | |  |  |  |  | 0.986 | 0.002 |  | 0.982 | 0.991 |
| **Likelihood ratio test of random intercept model vs. simple logistic model** | | | | | | **10-fold cross-validation adjusted ROC area,** Obs. 748^f^ | | | | |
| Chi^2^(2) 170.16, p-value<0.0001 | |  |  |  |  | 0.831 | 0.015 |  | 0.801 | 0.860 |
|  |  |  |  |  |  | **5-fold cross-validation adjusted ROC area,** Obs. 676^f^ | | | | |
|  |  |  |  |  |  | 0.821 | 0.016 |  | 0.789 | 0.853 |
|  |  |  |  |  |  | **Crude Global R^2^** | | 0.842 |  |  |
|  |  |  |  |  |  | **Conditional R^2^** | | 0.766 |  |  |

^a^ A three-level hierarchical logistic regression is shown. At the second and third level units, respectively, there are 510 households and 17 villages.

^b^ Estimate represents odds ratios for the fixed component and variance for the random component.

^c^ LN and EPG are abbreviations for natural log and eggs per gram, respectively.

^d^ Occupation represents income-earning work for each individual. The reference category for occupation is ‘no income-earning occupation’. This category included adults and children who did not work as well as housewives. For the full model, the global p-value equals 0.326 for occupation.

^e^ Six health workers were dropped from the regression as there was no variation in their drug receipt values; all health workers received praziquantel.

^f^ Observations were lost during cross-validation when unrealistic values for logistic regression were predicted, i.e. outside of the range of 0-1.

An explanation for the model in Table S7 can be found in the Methods section of the main text. Significant factors (p-value<0.05) for praziquantel receipt were infection intensity, home quality score, and water purification habits of the household whilst religion was borderline significantly associated (p-value=0.054) with drug receipt.

## Table S8: Main model for determinants of albendazole treatment receipt

|  | **A) Empty/variance components model**^a^ | | | | | **B) Random intercept model**^a^ | | | | | | |  |
| --- | --- | --- | --- | --- | --- | --- | --- | --- | --- | --- | --- | --- | --- |
| **Variable** | **Estimate**^b^ | **Std. err.** | **p-value** | **95% CI** | | **Estimate** | **Std. err.** | **p-value** | | **95% CI** | | | |
| **Fixed component** |  |  |  |  |  | **Fixed component** | | |  | |  | |  |
| Baseline LN(Hookworm EPG + 1) ^c^ |  |  |  |  |  | 1.011 | 0.077 | 0.889 | | 0.871 | | 1.173 | |
| Age |  |  |  |  |  | 1.014 | 0.013 | 0.299 | | 0.988 | | 1.041 | |
| Female |  |  |  |  |  | 1.520 | 0.608 | 0.296 | | 0.693 | | 3.330 | |
| Education |  |  |  |  |  | 0.987 | 0.065 | 0.842 | | 0.868 | | 1.122 | |
| Fisherman or fishmonger^d^ |  |  |  |  |  | 0.853 | 0.743 | 0.855 | | 0.155 | | 4.700 | |
| Business owner^d^ |  |  |  |  |  | 15.995 | 31.112 | 0.154 | | 0.353 | | 723.880 | |
| Rice farmer^d^ |  |  |  |  |  | 0.372 | 0.364 | 0.313 | | 0.055 | | 2.537 | |
| Other farmer^d^ |  |  |  |  |  | 1.732 | 0.939 | 0.311 | | 0.598 | | 5.012 | |
| School teacher^d^ |  |  |  |  |  | 0.065 | 0.121 | 0.140 | | 0.002 | | 2.442 | |
| Health worker^d^ |  |  |  |  |  | 14.332 | 43.793 | 0.384 | | 0.036 | | 5717.015 | |
| Other occupation^d^ |  |  |  |  |  | 4.669 | 4.777 | 0.132 | | 0.629 | | 34.678 | |
| Muslim household head |  |  |  |  |  | 0.215 | 0.124 | 0.007 | | 0.070 | | 0.663 | |
| Household head belongs to village majority tribe |  |  |  |  |  | 0.763 | 0.406 | 0.612 | | 0.269 | | 2.165 | |
| Total years household settled in village |  |  |  |  |  | 0.950 | 0.023 | 0.033 | | 0.906 | | 0.996 | |
| Home quality score |  |  |  |  |  | 1.254 | 0.096 | 0.003 | | 1.079 | | 1.457 | |
| Household purifies drinking water |  |  |  |  |  | 2.529 | 1.356 | 0.084 | | 0.884 | | 7.235 | |
| No home latrine |  |  |  |  |  | 0.052 | 0.049 | 0.002 | | 0.008 | | 0.331 | |
| Former or current village chairman in household |  |  |  |  |  | 16.679 | 15.471 | 0.002 | | 2.708 | | 102.739 | |
| Other former or current village government member in household |  |  |  |  |  | 10.208 | 9.865 | 0.016 | | 1.536 | | 67.849 | |
| Household seeks medical care from private clinics |  |  |  |  |  | 0.951 | 0.403 | 0.906 | | 0.414 | | 2.183 | |
| Village baseline hookworm prevalence |  |  |  |  |  | 0.909 | 0.016 | <0.001 | | 0.877 | | 0.941 | |
| Total homes in village |  |  |  |  |  | 0.997 | 0.003 | 0.291 | | 0.992 | | 1.002 | |
| Constant | 0.125 | 0.052 | <0.001 | 0.056 | 0.281 | 6.115 | 7.499 | 0.140 | | 0.553 | | 67.647 | |
| **Random component** |  |  |  |  |  | **Random component** | | |  | |  | |  |
| Household | 20.094 | 6.089 |  | 11.095 | 36.393 | 27.041 | 8.069 |  | | 15.067 | | 48.532 | |
| Village | 7.128 | 1.962 |  | 4.156 | 12.225 | 9.511 | 3.064 |  | | 5.058 | | 17.882 | |
| **Intraclass correlation** |  |  |  |  |  | **Intraclass correlation** | |  | |  | |  | |
| Household | 0.892 | 0.027 |  | 0.825 | 0.935 | 0.917 | 0.022 |  | | 0.863 | | 0.951 | |
| Village | 0.234 | 0.029 |  | 0.182 | 0.295 | 0.239 | 0.040 |  | | 0.169 | | 0.326 | |
|  | Obs. 935 |  |  |  |  | Obs. 935 |  |  | |  | |  | |
| **Likelihood ratio test of empty model vs. random intercept model** | | | |  |  | **ROC area,** Obs. 935 | |  | |  | |  | |
| Chi^2^(22) 50.95, p-value=0.0004 |  |  |  |  |  | 0.996 | 0.001 |  | | 0.994 | | 0.998 | |
| **Likelihood ratio test of random intercept model vs. simple logistic model** | | | | |  | **10-fold cross-validation adjusted ROC area,** Obs. 757^e^ | | | | | | |  |
| Chi^2^(2) 256.87, p-value<0.0001 |  |  |  |  |  | 0.866 | 0.014 |  | | 0.839 | | 0.893 | |
|  |  |  |  |  |  | **5-fold cross-validation adjusted ROC area,** Obs. 672^e^ | | | | | | |  |
|  |  |  |  |  |  | 0.865 | 0.015 |  | | 0.836 | | 0.893 | |
|  |  |  |  |  |  | **Crude Global R^2^** 0.846 | |  | |  | |  | |
|  |  |  |  |  |  | **Conditional R^2^** 0.918 | |  | |  | |  | |

^a^ A three-level hierarchical logistic regression is shown. At the second and third level units, respectively, there are 510 households and 17 villages.

^b^ Estimate represents odds ratios for the fixed component and variance for the random component.

^c^ LN and EPG are abbreviations for natural log and eggs per gram, respectively.

^d^ Occupation represents income-earning work for each individual. The reference category for occupation is ‘no income-earning occupation’. This category included adults and children who did not work as well as housewives. For the full model, the global p-value equals 0.380 for occupation.

^e^ Observations were lost during cross-validation when unrealistic values for logistic regression were predicted, i.e. outside of the range of 0-1.

An explanation for this model can be found in the Methods section of the main text. Religion, years in the village, home quality, and having no home latrine were significant (p-value<0.05) predictors of albendazole receipt. Also, having a former or current chairman or other village government member in the household was associated (p-value<0.05) with an increased likelihood of drug receipt. One village-level factor was significant (p-value<0.001) for albendazole receipt; individuals belonging to villages with high hookworm prevalence were less likely to receive treatment for hookworm infections.

## Table S9: Main model for determinants of albendazole receipt without health workers

|  | **Random intercept model** ^a^ | | | |  |
| --- | --- | --- | --- | --- | --- |
| **Variable** | **Estimate**^b^ | **Std. Err.** | **p-value** | **95% CI** | |
| **Fixed component** |  |  |  |  |  |
| Baseline LN(Hookworm EPG + 1) ^c^ | 1.015 | 0.080 | 0.854 | 0.869 | 1.185 |
| Age | 1.016 | 0.014 | 0.231 | 0.990 | 1.044 |
| Female | 1.937 | 0.764 | 0.094 | 0.894 | 4.196 |
| Education | 0.935 | 0.059 | 0.291 | 0.826 | 1.059 |
| Fisherman or fishmonger ^d^ | 0.918 | 0.785 | 0.921 | 0.172 | 4.901 |
| Business owner ^d^ | 1.913 | 2.488 | 0.618 | 0.150 | 24.480 |
| Rice farmer ^d^ | 0.251 | 0.266 | 0.192 | 0.031 | 2.001 |
| Other farmer ^d^ | 1.790 | 0.991 | 0.293 | 0.604 | 5.300 |
| School teacher ^d^ | 0.104 | 0.199 | 0.236 | 0.002 | 4.387 |
| Health worker | - | - | - | - | - |
| Other occupation ^d^ | 3.689 | 3.543 | 0.174 | 0.561 | 24.241 |
| Muslim household head | 0.229 | 0.123 | 0.006 | 0.080 | 0.655 |
| Household head belongs to village majority tribe | 1.153 | 0.528 | 0.756 | 0.470 | 2.828 |
| Total years household settled in village | 0.943 | 0.018 | 0.002 | 0.908 | 0.979 |
| Home quality score | 1.260 | 0.098 | 0.003 | 1.081 | 1.469 |
| Household purifies drinking water | 1.376 | 0.595 | 0.461 | 0.590 | 3.211 |
| No home latrine | 0.039 | 0.038 | 0.001 | 0.006 | 0.258 |
| Former or current village chairman in household | 9.383 | 8.651 | 0.015 | 1.540 | 57.168 |
| Other former or current village government member in household | 12.390 | 13.590 | 0.022 | 1.444 | 106.346 |
| Household seeks medical care from private clinics | 0.975 | 0.411 | 0.952 | 0.426 | 2.230 |
| Village baseline hookworm prevalence | 0.905 | 0.016 | <0.001 | 0.874 | 0.936 |
| Total homes in village | 0.999 | 0.003 | 0.648 | 0.993 | 1.004 |
| Constant | 3.886 | 4.582 | 0.250 | 0.385 | 39.198 |
| **Random component** |  |  |  |  |  |
| Household | 26.220 | 7.397 |  | 15.083 | 45.579 |
| Village | 11.958 | 3.788 |  | 6.426 | 22.250 |
| **Intraclass correlation** |  |  |  |  |  |
| Household | 0.921 | 0.019 |  | 0.875 | 0.951 |
| Village | 0.288 | 0.060 |  | 0.186 | 0.418 |
| Obs. 929 |  |  |  |  |  |
| **ROC area** | 0.996 | 0.001 |  | 0.993 | 0.998 |
| **Crude Global R^2^** | 0.844 |  |  |  |  |
| **Conditional R^2^** | 0.921 |  |  |  |  |

^a^ A three-level hierarchical logistic regression is shown. At the second and third level units, respectively, there are 510 households and 17 villages.

^b^ Estimate represents odds ratios for the fixed component and variance for the random component.

^c^ LN and EPG are abbreviations for natural log and eggs per gram, respectively.

^d^ Occupation represents income-earning work for each individual. The reference category for occupation is ‘no income-earning occupation’. This category included adults and children who did not work as well as housewives. The global p-value equals 0.294 for occupation.

This table is presented as a robustness check to the results shown in Supplementary Table S8. This model is specified in the same manner as Table S8; only health workers—six observations—are excluded. These observations are excluded to enable a comparison with the main model for praziquantel receipt (Table S7). In the praziquantel model, health workers were excluded because there was no variation in praziquantel receipt. All determinants for albendazole receipt remain significant (p-value<0.05) and in the same direction when health workers were removed from the regression.

## Table S10: Main model for determinants of praziquantel receipt without *S. mansoni* infection intensity

|  | **Random intercept model** ^a^ | | | |  |
| --- | --- | --- | --- | --- | --- |
| **Variable** | **Estimate** ^b^ | **Std. Err.** | **p-value** | **95% CI** | |
| **Fixed component** |  |  |  |  |  |
| Age | 1.007 | 0.009 | 0.483 | 0.988 | 1.025 |
| Female | 0.845 | 0.239 | 0.551 | 0.486 | 1.469 |
| Education | 0.931 | 0.045 | 0.137 | 0.847 | 1.023 |
| Fisherman or fishmonger ^c^ | 1.293 | 0.899 | 0.711 | 0.331 | 5.054 |
| Business owner ^c^ | 1.038 | 0.808 | 0.962 | 0.226 | 4.771 |
| Rice farmer ^c^ | 0.192 | 0.167 | 0.058 | 0.035 | 1.061 |
| Other farmer ^c^ | 1.618 | 0.659 | 0.238 | 0.728 | 3.596 |
| School teacher ^c^ | 1.213 | 1.511 | 0.877 | 0.106 | 13.934 |
| Health worker ^d^ | - | - | - | - | - |
| Other occupation ^c^ | 1.825 | 1.297 | 0.397 | 0.453 | 7.351 |
| Muslim household head | 0.474 | 0.179 | 0.048 | 0.227 | 0.993 |
| Household head belongs to village majority tribe | 2.070 | 0.672 | 0.025 | 1.096 | 3.913 |
| Total years household settled in village | 0.998 | 0.014 | 0.876 | 0.971 | 1.026 |
| Home quality score | 1.182 | 0.061 | 0.001 | 1.068 | 1.309 |
| Household purifies drinking water | 2.130 | 0.761 | 0.034 | 1.058 | 4.290 |
| No home latrine | 0.330 | 0.233 | 0.116 | 0.083 | 1.313 |
| Former or current village chairman in household | 4.124 | 3.312 | 0.078 | 0.855 | 19.902 |
| Other former or current village government member in household | 1.724 | 1.016 | 0.355 | 0.543 | 5.473 |
| Household seeks medical care from private clinics | 0.863 | 0.286 | 0.657 | 0.450 | 1.654 |
| Village baseline *S. mansoni* prevalence | 1.007 | 0.020 | 0.737 | 0.968 | 1.048 |
| Total homes in village | 0.996 | 0.005 | 0.421 | 0.985 | 1.006 |
| Constant | 0.526 | 0.663 | 0.610 | 0.044 | 6.221 |
| **Random component** |  |  |  |  |  |
| Household | 7.755 | 2.144 |  | 4.511 | 13.333 |
| Village | 2.693 | 1.217 |  | 1.111 | 6.529 |
| **Intraclass correlation** |  |  |  |  |  |
| Household | 0.761 | 0.050 |  | 0.649 | 0.845 |
| Village | 0.196 | 0.064 |  | 0.099 | 0.352 |
| Obs. 929 ^d^ |  |  |  |  |  |
| **ROC area** | 0.986 | 0.003 |  | 0.981 | 0.991 |
| **Crude Global R^2^** | 0.840 |  |  |  |  |
| **Conditional R^2^** | 0.763 |  |  |  |  |

^a^ A three-level hierarchical logistic regression is shown. At the second and third level units, respectively, there are 510 households and 17 villages.

^b^ Estimate represents odds ratios for the fixed component and variance for the random component.

^c^ Occupation represents income-earning work for each individual. The reference category for occupation is ‘no income-earning occupation’. This category included adults and children who did not work as well as housewives. The global p-value equals 0.332 for occupation.

^d^ Six health workers were dropped from the regression as there was no variation in their drug receipt values; all health workers received praziquantel.

The model presented here is specified in the same manner as the model in Supplementary Table S7 except individual S. mansoni infection intensity was excluded. This regression is presented as a robustness check against the significant factors of drug receipt. This variable was excluded in the event that the direction of the relationship between treatment and infection status is confounded, i.e. the dependent variable of drug receipt affects the infection intensity of an individual. Infection intensity will be an indication of past treatment receipt. After removing infection intensity, all factors previously found (Table S7) to be determinants of praziquantel receipt remained significant (p-value<0.05) and in these effects were in the same direction.

## Table S11: Main model for determinants of albendazole receipt without hookworm infection intensity

|  | **Random intercept model** ^a^ | | | |  |
| --- | --- | --- | --- | --- | --- |
| **Variable** | **Estimate**^b^ | **Std. err.** | **p-value** | **95% CI** | |
| **Fixed component** |  |  |  |  |  |
| Age | 1.017 | 0.014 | 0.199 | 0.991 | 1.044 |
| Female | 1.947 | 0.765 | 0.090 | 0.901 | 4.205 |
| Education | 0.935 | 0.059 | 0.291 | 0.826 | 1.059 |
| Fisherman or fishmonger ^c^ | 0.893 | 0.766 | 0.895 | 0.166 | 4.798 |
| Business owner ^c^ | 1.855 | 2.432 | 0.637 | 0.142 | 24.230 |
| Rice farmer ^c^ | 0.258 | 0.271 | 0.197 | 0.033 | 2.021 |
| Other farmer ^c^ | 1.771 | 0.980 | 0.302 | 0.599 | 5.241 |
| School teacher ^c^ | 0.094 | 0.184 | 0.227 | 0.002 | 4.378 |
| Health worker ^c^ | 13.126 | 39.506 | 0.392 | 0.036 | 4787.044 |
| Other occupation ^c^ | 3.656 | 3.533 | 0.180 | 0.550 | 24.297 |
| Muslim household head | 0.227 | 0.121 | 0.005 | 0.080 | 0.643 |
| Household head belongs to village majority tribe | 1.124 | 0.519 | 0.801 | 0.454 | 2.778 |
| Total years household settled in village | 0.941 | 0.018 | 0.002 | 0.907 | 0.978 |
| Home quality score | 1.266 | 0.101 | 0.003 | 1.082 | 1.481 |
| Household purifies drinking water | 1.400 | 0.605 | 0.436 | 0.600 | 3.267 |
| No home latrine | 0.037 | 0.036 | 0.001 | 0.006 | 0.250 |
| Former or current village chairman in household | 9.638 | 8.962 | 0.015 | 1.558 | 59.638 |
| Other former or current village government member in household | 13.136 | 14.781 | 0.022 | 1.448 | 119.185 |
| Household seeks medical care from private clinics | 0.999 | 0.424 | 0.999 | 0.435 | 2.297 |
| Village baseline hookworm prevalence | 0.905 | 0.015 | <0.001 | 0.875 | 0.935 |
| Total homes in village | 0.999 | 0.003 | 0.641 | 0.993 | 1.004 |
| Constant | 3.812 | 4.496 | 0.257 | 0.378 | 38.466 |
| **Random component** |  |  |  |  |  |
| Household | 26.807 | 7.429 |  | 15.573 | 46.147 |
| Village | 12.052 | 3.793 |  | 6.504 | 22.333 |
| **Intraclass correlation** |  |  |  |  |  |
| Household | 0.922 | 0.018 |  | 0.878 | 0.951 |
| Village | 0.286 | 0.059 |  | 0.185 | 0.414 |
| Obs. 935 |  |  |  |  |  |
| **ROC area** | 0.996 | 0.001 |  | 0.994 | 0.998 |
| **Crude Global R^2^** | 0.845 |  |  |  |  |
| **Conditional R^2^** | 0.922 |  |  |  |  |

^a^ A three-level hierarchical logistic regression is shown. At the second and third level units, respectively, there are 510 households and 17 villages.

^b^ Estimate represents odds ratios for the fixed component and variance for the random component.

^c^ Occupation represents income-earning work for each individual. The reference category for occupation is ‘no income-earning occupation’. This category included adults and children who did not work as well as housewives. The global p-value equals 0.344 for occupation.

The model presented here is specified in the same manner as the model in Supplementary Table S8 except individual hookworm infection intensity was excluded. This regression is presented as a robustness check against the significant factors of drug receipt. This variable was excluded in the event that the direction of the relationship between treatment and infection status is confounded, i.e. the dependent variable of drug receipt affects the infection intensity of an individual. Infection intensity will be an indication of past treatment receipt. After removing infection intensity, all factors previously found (Table S8) to be determinants of albendazole receipt remained significant (p-value<0.05) and in these effects were in the same direction.

## Table S12: Univarate regressions for praziquantel and albendazole receipt

|  | **A) Dependent variable: praziquantel receipt** ^a^ | | | | | **B) Dependent variable: albendazole receipt** ^a^ | | | | |
| --- | --- | --- | --- | --- | --- | --- | --- | --- | --- | --- |
| **Predictors in univariate regressions** ^a^ | **Odds ratio** | **Std. err** | **p-value** | **95% CI** |  | **Odds ratio** | **Std. err** | **p-value** | **95% CI** |  |
| Baseline LN (*S. mansoni* EPG +1) ^b^ | 0.856 | 0.047 | 0.005 | 0.768 | 0.953 |  |  |  |  |  |
| Baseline LN(Hookworm EPG + 1) ^b^ | |  |  |  |  | 1.023 | 0.068 | 0.738 | 0.897 | 1.166 |
| Age | 1.009 | 0.007 | 0.208 | 0.995 | 1.022 | 1.022 | 0.009 | 0.010 | 1.005 | 1.040 |
| Female | 0.783 | 0.210 | 0.362 | 0.462 | 1.326 | 1.019 | 0.328 | 0.953 | 0.543 | 1.913 |
| Education | 0.974 | 0.040 | 0.516 | 0.898 | 1.056 | 1.000 | 0.049 | 0.995 | 0.908 | 1.101 |
| Fisherman or fishmonger | 0.984 | 0.612 | 0.979 | 0.291 | 3.327 | 1.069 | 0.757 | 0.925 | 0.267 | 4.285 |
| Business owner | 0.894 | 0.640 | 0.876 | 0.220 | 3.639 | 4.710 | 4.374 | 0.095 | 0.763 | 29.072 |
| Rice farmer | 0.246 | 0.199 | 0.083 | 0.050 | 1.201 | 0.448 | 0.391 | 0.358 | 0.081 | 2.482 |
| Other farmer | 1.797 | 0.573 | 0.066 | 0.962 | 3.359 | 2.854 | 1.181 | 0.011 | 1.269 | 6.421 |
| School teacher | 0.882 | 1.004 | 0.912 | 0.095 | 8.206 | 0.259 | 0.496 | 0.481 | 0.006 | 11.090 |
| Health worker ^c^ | - | - | - | - | - | 6.069 | 12.572 | 0.384 | 0.105 | 351.911 |
| Other occupation | 1.684 | 1.104 | 0.427 | 0.466 | 6.084 | 4.366 | 3.731 | 0.085 | 0.818 | 23.305 |
| Muslim household head | 0.551 | 0.205 | 0.109 | 0.266 | 1.142 | 0.493 | 0.215 | 0.105 | 0.210 | 1.160 |
| Household head belongs to village majority tribe | 2.097 | 0.651 | 0.017 | 1.141 | 3.853 | 1.262 | 0.493 | 0.551 | 0.587 | 2.713 |
| Total years household settled in village | 1.016 | 0.013 | 0.213 | 0.991 | 1.043 | 0.984 | 0.016 | 0.339 | 0.953 | 1.017 |
| Home quality score | 1.199 | 0.058 | <0.001 | 1.090 | 1.318 | 1.242 | 0.079 | 0.001 | 1.097 | 1.407 |
| Household purifies drinking water | 2.189 | 0.780 | 0.028 | 1.089 | 4.402 | 3.146 | 1.438 | 0.012 | 1.284 | 7.708 |
| No home latrine | 0.256 | 0.180 | 0.052 | 0.065 | 1.014 | 0.287 | 0.244 | 0.142 | 0.054 | 1.518 |
| Former or current village chairman in household | 5.518 | 4.415 | 0.033 | 1.150 | 26.472 | 6.231 | 6.304 | 0.071 | 0.858 | 45.262 |
| Other former or current village government member in household | 2.422 | 1.405 | 0.127 | 0.777 | 7.549 | 4.444 | 3.492 | 0.058 | 0.953 | 20.728 |
| Household seeks medical care from private clinics | 1.172 | 0.385 | 0.628 | 0.616 | 2.232 | 0.943 | 0.368 | 0.881 | 0.439 | 2.026 |
| Village baseline *S. mansoni* prevalence | 0.997 | 0.019 | 0.866 | 0.960 | 1.035 |  |  |  |  |  |
| Village baseline hookworm prevalence | |  |  |  |  | 0.868 | 0.022 | <0.001 | 0.826 | 0.912 |
| Total homes in village | 0.995 | 0.005 | 0.301 | 0.985 | 1.005 | 1.012 | 0.008 | 0.132 | 0.996 | 1.028 |
|  | Obs. 929 ^c^ |  |  |  |  | Obs. 935 |  |  |  |  |

^a^ A three-level hierarchical logistic regression is shown. At the second and third level units, respectively, there are 510 households and 17 villages. Constants and random effects are not shown. Single regressions are shown with one predictor.

^b^ LN and EPG are abbreviations for natural log and eggs per gram, respectively.

^c^ Six health workers were dropped from the regression as there was no variation in their drug receipt values; all health workers received praziquantel.

Unadjusted effects for each variable are presented. Each variable represents a single regression for either praziquantel or albendazole receipt. Although stepwise or forward selection was not used to select the predictors, all variables that were found significant in the main text were significant if they would have been chosen by forward selection (p-value<0.15).

## References

1. Kabatereine N, Standley C, Sousa-Figueiredo J, et al. Integrated prevalence mapping of schistosomiasis, soil-transmitted helminthiasis and malaria in lakeside and island communities in Lake Victoria, Uganda. Parasit Vectors. 2011;4:232.

2. Kabatereine N, Vennervald B, Ouma J, et al. Adult resistance to schistosomiasis mansoni: age-dependence of reinfection remains constant in communities with diverse exposure patterns. Parasitology. 1999;118:101-5.

3. Tukahebwa E, Magnussen P, Madsen H, et al. A Very High Infection Intensity of Schistosoma mansoni in a Ugandan Lake Victoria Fishing Community Is Required for Association with Highly Prevalent Organ Related Morbidity. PLoS Negl Trop Dis. 2013;7:e2268.

4. Fitzsimmons C, Joseph S, Jones F, et al. Chemotherapy for schistosomiasis in Ugandan fishermen: treatment can cause a rapid increase in interleukin-5 levels in plasma but decreased levels of eosinophilia and worm-specific immunoglobulin E. Infect Immun. 2004;72:4023-30.

5. Pullan R, Kabatereine N, Quinnell R, Brooker S. Spatial and Genetic Epidemiology of Hookworm in a Rural Community in Uganda. PLoS Negl Trop Dis. 2010;4:e713.

6. Cattaneo MD. Efficient semiparametric estimation of multi-valued treatment effects under ignorability. J Econometrics. 2010;155:138-54.

7. Emsley R, Lunt M, Pickles A, Dunn G. Implementing double-robust estimators of causal effects. Stata J. 2008;8:334-53.

8. Williams RL. A note on robust variance estimation for cluster-correlated data. Biometrics. 2000;56:645-6.
